# Contrasting selective patterns across the segmented genome of bluetongue virus in a global reassortment hotspot

**DOI:** 10.1093/ve/vez027

**Published:** 2019-08-05

**Authors:** Maude Jacquot, Pavuluri P Rao, Sarita Yadav, Kyriaki Nomikou, Sushila Maan, Y Krishna Jyothi, Narasimha Reddy, Kalyani Putty, Divakar Hemadri, Karam P Singh, Narender Singh Maan, Nagendra R Hegde, Peter Mertens, Roman Biek

**Affiliations:** 1 Institute of Biodiversity, Animal Health and Comparative Medicine, Boyd Orr Centre for Population and Ecosystem Health, College of Medical, Veterinary and Life Sciences, University of Glasgow, Glasgow, UK; 2 Ella Foundation, Genome Valley Hyderabad, Hyderabad, Telangana, India; 3 The Pirbright Institute, Pirbright, Woking, Surrey, UK; 4 MRC—University of Glasgow Centre for Virus Research, Glasgow, UK; 5 College of Veterinary Sciences, LLR University of Veterinary and Animal Sciences, Hisar, Haryana, India; 6 Veterinary Biological and Research Institute, Vijayawada, Andhra Pradesh, India; 7 PVNR Telangana Veterinary University, Hyderabad, Telangana, India; 8 ICAR—National Institute of Veterinary Epidemiology and Disease Informatics, Bengaluru, Karnataka, India; 9 Centre for Animal Disease Research and Diagnosis, Division of Pathology, ICAR—Indian Veterinary Research Institute, Izatnagar, Bareilly, Uttar Pradesh, India; 10 The School of Veterinary Medicine and Science, University of Nottingham, Sutton Bonington, Leicestershire, UK

**Keywords:** bluetongue virus, evolution, reassortment, India, selection

## Abstract

For segmented viruses, rapid genomic and phenotypic changes can occur through the process of reassortment, whereby co-infecting strains exchange entire segments creating novel progeny virus genotypes. However, for many viruses with segmented genomes, this process and its effect on transmission dynamics remain poorly understood. Here, we assessed the consequences of reassortment for selection on viral diversity through time using bluetongue virus (BTV), a segmented arbovirus that is the causative agent of a major disease of ruminants. We analysed ninety-two BTV genomes isolated across four decades from India, where BTV diversity, and thus opportunities for reassortment, are among the highest in the world. Our results point to frequent reassortment and segment turnover, some of which appear to be driven by selective sweeps and serial hitchhiking. Particularly, we found evidence for a recent selective sweep affecting segment 5 and its encoded NS1 protein that has allowed a single variant to essentially invade the full range of BTV genomic backgrounds and serotypes currently circulating in India. In contrast, diversifying selection was found to play an important role in maintaining genetic diversity in genes encoding outer surface proteins involved in virus interactions (VP2 and VP5, encoded by segments 2 and 6, respectively). Our results support the role of reassortment in driving rapid phenotypic change in segmented viruses and generate testable hypotheses for *in vitro* experiments aiming at understanding the specific mechanisms underlying differences in fitness and selection across viral genomes.

## 1. Introduction

RNA viruses have an extraordinary ability to diversify and evolve. The observed high evolution rate of RNA viruses is partly attributed to limited or no proof reading capacity of RNA-dependent RNA polymerases. In addition, for viruses with segmented genomes, rapid genomic and phenotypic changes can occur through the process of reassortment, whereby co-infecting different viral strains exchange entire segments (see McDonald et al. 2016 for a review). This has received considerable research attention in certain groups, such as influenza viruses, where reassortment has been linked to changes in host range and the emergence of novel strains (e.g. Li et al. 2004; Schweiger, Bruns, and Meixenberger 2006; Simonsen et al. 2007; Nelson et al. 2008; Rambaut et al. 2008; Smith et al. 2009; Trifonov et al. 2009; Wu et al. 2015; Pohlmann et al. 2017). However, for many segmented-RNA viruses, in particular those affecting non-human hosts, the consequences of reassortment in relation to selection and the maintenance of viral diversity at the genome scale remain poorly understood.

Bluetongue virus (BTV) belongs to the genus *Orbivirus* of the family *Reoviridae*. It is responsible for an economically important disease of ruminants, bluetongue disease (BT). The distribution of BTV overlaps with the distribution of BTV transmission-competent midges belonging to the genus *Culicoides* (Tabachnick 2004). BT is endemic in most parts of tropical and subtropical regions, and seasonal incursions of the disease are observed in parts of temperate regions during the summer (Mellor and Wittmann 2002; Purse et al. 2005; Mellor et al. 2008). Bluetongue outbreaks cause severe economic damage due to the direct effects on livestock, trade restrictions, and the cost of surveillance and control (e.g. Osburn 1994; Maclachlan et al. 2009; Velthuis et al. 2010; Pinior et al. 2015). In the USA for example, BTV is thought to cause losses in the order of US$125 million annually (Tabachnick 1996).

The genome of BTV consists of ten double stranded RNA (dsRNA) segments with each segment coding for at least one major protein. Some of these proteins form part of the three-layered structure of the virus whereas others are non-structural. The inner capsid consists of the three minor viral proteins (VP) required for genome transcription and replication, i.e. VP1 (RNA-dependent RNA polymerase), VP4 (RNA capping, guanylyltransferase, transmethylase 1 and 2), and VP6 (RNA helicase), coded by genome Segments 1, 4, and 9, respectively. These proteins, together with the ten genome segments, are encased by a shell composed of VP3 encoded by Segment 3, which is surrounded by the intermediate capsid protein layer composed of VP7 coded by Segment 7. The outer capsid is made up of VP2 and VP5, coded by Segments 2 and 6, respectively. VP2 and VP5 are involved in the virus binding to and entering into the mammalian host (and likely also insect vector) cells (Mertens and Diprose 2004; Lourenco and Roy 2011). Variability in the sequence of VP2, and to some extent VP5, contributes to the specificity of differential neutralising antibody responses in infected animals, and forms the basis for classifying BTV into at least twenty-seven different serotypes. Five non-structural proteins (NS) are produced by BTV in infected cells. NS1 enhances VP synthesis and is involved in formation of tubules in the cytoplasm of infected cells (Boyce, Celma, and Roy 2012), while NS2 is a major component of viral inclusion bodies present in infected cells (Butan and Tucker 2010). These two proteins are coded by genome Segments 5 and 8, respectively. Segment 10 codes for NS3, a glycoprotein involved in egress of the virus (particularly from insect cells), and NS3a, a truncated version of NS3 produced by an alternate in-frame start codon (Wu et al. 1992; Han and Harty 2004; Wirblich, Bhattacharya, and Roy 2006; Celma and Roy 2009; Bhattacharya and Roy 2013). NS4 is a small peptide of 77–79 residues coded by Segment 9 from an alternate open reading frame (ORF) from that of VP6 (Belhouchet et al. 2011; Ratinier et al. 2011). A second alternate ORF was also detected in Segment 10 of different orbiviruses (including BTV) coding for a small peptide (50–59 aa), a putative NS5 protein (Stewart et al. 2015).

The segmented nature of the BTV genome can lead to the generation of a mosaic of viruses through independent reassortment of different genome segments (Oberst et al. 1987; Samal et al. 1987; Batten et al. 2008; Rambaut et al. 2008; Shaw et al. 2013; Nomikou et al. 2015; Schirtzinger et al. 2018). While this has the potential to change the phenotypic characteristics of the virus, as seen in other segmented viruses (e.g. Li et al. 2004; Schweiger, Bruns, and Meixenberger 2006; Simonsen et al. 2007; Nelson et al. 2008; Rambaut et al. 2008; Smith et al. 2009; Trifonov et al. 2009; Wu et al. 2015; Pohlmann et al. 2017), conclusive examples of this have been lacking for BTV. *In vitro*, BTV reassortment appears to be a flexible process that is largely unaffected by selective constraints (Shaw et al. 2013). In contrast, a recent analysis of BTV field strains from Europe revealed non-random associations between some segments and found a higher probability of amino acid substitutions on tree branches associated with reassortment events (Nomikou et al. 2015). While these latter results support a more important role for reassortment affecting BTV genome evolution under field conditions, there is a need for further studies aimed at assessing its significance for driving adaptive and epidemiological dynamics *in vivo*.

Reassortment is expected to be most common and biologically relevant in areas supporting a high diversity of BTV serotypes and strains. Serological data show that twenty-two BTV serotypes exist in India, making it a region with one of the highest levels of BTV diversity in the world (Rao et al. 2016; Maan et al. 2017). Although most of these serotypes appear to be ‘native’ to the Indian sub-continent, there is evidence for the introduction of exotic BTV serotypes and strains belonging to the major western topotype, from Africa and the USA, linked to the use of live vaccines or the livestock trade (Maan et al. 2015; Krishnajyothi et al. 2016), and contributing to further diversity. In addition, the severity of BT outbreaks in Indian sheep has increased significantly in recent decades (Prasad et al. 2009; Chand et al. 2015; Rao et al. 2016), raising the possibility of selection of more virulent (possibly reassortant) strains and recent evolutionary changes in the virus driving rapid phenotypic changes. Together, this makes India an important area for studying BTV evolution and its epidemiological implications. However, studies examining the evolutionary history of BTV in India based on whole genome data have begun only recently (Maan et al. 2015) and have so far been limited in scope.

Here, we triple the number of BTV genome sequences available from India (from 30 to >90), based on samples collected over the last four decades. Using these data, our aim was (1) to quantify the processes of reassortment and selection, and (2) to determine how these processes are interacting to shape the evolution of the BTV genome in a setting characterised by a high diversity of circulating strains.

## 2. Material and methods

### 2.1 Cells and virus isolates

Fifty-seven BTV isolates from India were newly sequenced for this study (Supplementary Table S1). These isolates were obtained from ethylenediamine tetraacetic acid blood or tissue samples (spleen) collected from outbreaks from animals (sheep, goat) showing clinical signs of BT between 1993 and 2015.

For forty-six isolates, *Culicoides sonorensis* cells (KC cells), an insect cell line derived from the embryo of *C*. *sonorensis*, BHK-21 (clone 13), and BSR cells (a sub-clone of BHK-21 cells) were used for isolation of BTV. KC cells were infected with washed and lysed red blood cells or homogenised tissues. KC viral isolates were propagated in BHK or BSR cell monolayers (175 cm^2^ flasks) at 37 °C in Dulbecco's modified Eagle's medium supplemented with 100 units/ml of penicillin and 100 μg/ml streptomycin until completion of cytopathic effects, generally between 48 and 72 h post-infection and passaged 3–6 times in BHK or BSR cells before RNA isolation.

For eleven isolates, BHK-21 cells were infected with different isolates of BTV and the lysed cells were pelleted after completion of cytopathic effects.

### 2.2 RNA isolation

BTV dsRNA was extracted from pellets of virus-infected cell cultures using a guanidinium isothiocyanate procedure as previously described (Attoui, Billoir, and Cantaloube 2000). Total RNA was extracted from the infected cell pellet using TRIZOL (Invitrogen, USA) as per manufacturer’s instructions. Cellular single stranded RNA was separated from the viral dsRNA by precipitating in 2 M lithium chloride (LiCl) at 4 °C overnight. An equal volume of isopropanol and 0.25 volumes of 7.5 M ammonium acetate were added to precipitate dsRNA at −20 °C for 2 h, pellets were washed with 70 per cent ethanol and finally suspended in 50 µl of nuclease free water. The isolated dsRNA was shipped to MRC-CVR, UK for deep sequencing. For the eleven BHK isolates, total RNA was isolated from the pellet by acid phenol method (Gauthami et al. 2015).

### 2.3 Sequencing

Full-length cDNA copies of BTV genome segments were synthesised and amplified in a sequence-independent manner using the ‘anchor spacer-ligation’ method (Maan et al. 2007). BTV dsRNA was DNase treated using Turbo DNase (Ambion Life Technologies), according to manufacturer’s instructions and ligated to an anchor primer (Maan et al. 2007). After purification of ligated RNA using the RNA Clean & Concentrator kit (Zymo research, USA), purified RNA was reverse transcribed to generate full length cDNA using SuperScript III first-strand synthesis system (Thermo Fisher Scientific). Amplification of the resulting cDNA was performed using KAPA HiFi Hot Start Ready Mix (Kapa Biosystems, UK) using the ‘5-15-1’ primer, which is complementary to the anchor primer followed by purification of dsDNA PCR products using the DNA Clean & Concentrator kit (Zymo research). Quantity and quality of PCR products were tested using Qubit Fluorimeter 3 (Life Technologies Q32854) and Agilent 4200 TapeStation (Agilent Technologies, 5067-5584) and the concentration of each sample was adjusted to 0.2 ng/μl for library preparation using the Illumina Nextera XT DNA library preparation kit. The amplified PCR reaction products were purified using Ampure XP beads (Agencourt, Beckman Coulter). The concentration of library DNA was measured using Qubit Fluorometer 3 and Qubit dsDNA high sensitivity (HS) Assay Kit (Life Technologies, Q32854). The library sizing (quality and distribution) and quantification were determined using the High Sensitivity D5000 Screen Tape assay (Agilent, 5067-5588) in a 4200 TapeStation System (Agilent). The library pool (at 4 nmole) was loaded in the MiSeq reagent kit Version 2 cartridge (Illumina) and paired-end reads of 150 bp were produced on a MiSeq platform. The quality of the resultant fastq files were assessed using FastQC (http://www.bioinformatics.babraham.ac.uk/projects/fastqc/, accessed 1 July 2019). Primers, Illumina adapter sequences, low quality-bases on the 3′ end of reads with Phred-scaled quality less than 30 and reads with a length <50 bp were removed using Trim Galore (https://www.bioinformatics.babraham.ac.uk/projects/trim_galore/, accessed 1 July 2019).

For the BHK-21 isolates, genomic libraries were prepared, sequenced, and processed as described in Rao et al. (2013).

### 2.4 Genome sequence reconstruction

Whole genomes were generated using *de novo* assembly and reference-based mapping methods.

Briefly, quality-controlled reads were *de novo* assembled using the SPAdes genome assembler version: 3.10.1 (Bankevich et al. 2012). Resulting contigs were grouped by segment and in each case, the one covering the largest portion of the segment was retained. For reference-based mapping, a unique reference was selected for each sample based on the best match from a BLAST search for each segment. Quality-controlled reads were mapped against the reference sequences using Tanoti (http://bioinformatics.cvr.ac.uk/tanoti.php, accessed 1 July 2019) and Bowtie2 (Langmead and Salzberg 2012). Assemblies were viewed using UGENE (Okonechnikov, Golosova, and Fursov 2012). Summary statistics of mapped and unmapped reads, depth and breadth of coverage across all genome segments for all samples are shown in Supplementary Table S2. Consensus sequences were generated using SAMtools (Li et al. 2009). Sequences generated in this study have been submitted in GenBank under accession numbers listed in Supplementary Table S1.

### 2.5 Sequence alignments

The consensus sequences generated for individual genome segments were combined with sequences from one Pakistani and other Indian BTV genomes available from GenBank in November 2017 (and for which metadata was sufficient), bringing the total number of genomes used in this study to ninety-two. Spatial distribution of samples and other metadata are summarised in Supplementary Fig. S1 and Table S1.

For each segment, the main coding region—VP1, VP2, VP3, VP4, NS1, VP5, VP7, NS2, VP6, and NS3 for the segments 1–10, respectively—was aligned using PAL2NAL (Suyama, Torrents, and Bork 2006) according to protein sequences aligned with Clustal Omega (Sievers et al. 2011). For the remainder of the manuscript, these coding regions are referred as Seg-1 to Seg-10. Alignments were also obtained for NS4 and NS5 (alternate ORFs on Seg-9 and Seg-10) but were only used for selection pattern analyses, as described below.

### 2.6 Phylogenies and evolutionary rate estimates

For each BTV segment, we evaluated the temporal signal of the heterochronous sequences with TempEst v1.5.1 (Rambaut et al. 2016) and time-scaled Bayesian phylogenetic trees were estimated in BEAST 1.8.4 (Drummond et al. 2012) using the SDR06 codon substitution model for the coding sequence (Shapiro, Rambaut, and Drummond 2006) under a relaxed molecular clock with a lognormal distribution of rates. A Gaussian Markov Random Field Bayesian skyride coalescent model was used as the tree prior (Minin, Bloomquist, and Suchard 2008). For samples for which exact dates of collection were unavailable, approximate dates were used and uncertainty added to the tips (Precision column in Supplementary Table S1). For each segment, a Monte Carlo Markov chain (MCMC) was run for the number of generations needed to achieve stationary distributions and convergence (5 × 10^8^–5 × 10^9^ generations) and sampling frequency adjusted accordingly to yield 10,000 samples from the posterior. We used Tracer v1.6 (Rambaut et al. 2013) to visualise the posterior distribution of each parameter and to obtain an estimate of the effective sample size (ESS) after removal of the initial 10 per cent burn-in. We assumed a run had reached sufficient mixing if the ESS of all the parameters was above 200. For each BTV segment, a maximum clade credibility (MCC) tree was produced after removal of the initial 10 per cent burn-in based on common ancestor heights method (Heled and Bouckaert 2013) using the auxiliary program TreeAnnotator included in the BEAST package.

### 2.7 Sequence clustering and reassortment

In order to quantify functional genetic diversity present in Indian BTV genomes and its changes through time, we used the Bayesian clustering algorithm provided by the program K-Pax2 (Pessia et al. 2015). This approach overcomes problems encountered with standard clustering tools relying on arbitrary criteria that are challenging to calibrate in cases where evolutionary histories and levels of divergence may vary considerably across the genome. For each segment, clusters of sequences based on shared amino acid changes were defined via K-Pax2. Ten independent runs were carried out from different starting configurations under the default prior settings and upper bound values for the number of clusters corresponding to the number of samples (*n* = 92). The optimal clustering of each segment was identified using the log posterior scoring function of the method. Delineated clusters were highlighted on the plotted phylogenies using the R package ggtree (Yu et al. 2017).

We used multi-dimensional scaling (MDS) plots summarising tree-to-tree variation in branch lengths to assess how reassortment might have broken up associations among segments (Rambaut et al. 2008; Bahl et al. 2013). For each BTV segment, 500 trees sampled from the MCMC chain were used to determine the time to most recent common ancestor (TMRCA) for any pair of Indian isolates sampled within the same year (from 2003 onwards). The correlation coefficient of TMRCA estimates across all pairwise comparisons of trees was calculated and used to estimate the tree-to-tree distance (inverse). The matrix of tree-to-tree distances was then plotted in two dimensions using MDS. In this analysis, the spread of each point cloud represents the statistical uncertainty in the phylogenetic history of each BTV segment, where overlapping point clouds represent cases for which the null hypothesis of no reassortment cannot be rejected.

Reassortment events were further identified by the graph incompatibility-based reassortment finder (GiRaF) program originally developed for influenza viruses (Nagarajan and Kingsford 2011). Fifty repeats of the following two-steps procedure were performed. First, 1,000 unrooted candidate trees were inferred for each segment with MrBayes v3.2.7 (Ronquist and Huelsenbeck 2003; Ronquist et al. 2012) using a GTR+I+G substitution model, a burn-in of 50 per cent (100,000 iterations), and sampling every 200 iterations. This tree sample was used to account for uncertainty in the phylogenetic inference for each segment. GiRaF, which identifies sets of taxa with different phylogenetic placement across the trees, was applied to each tree set using default settings across all forty-five possible combinations of segments to comprehensively catalogue reassortment events.

We also used Recombination Detection Program (RDP) version 4.97 (Martin et al. 2015) with default settings, except that we invoked the ‘scan for reassortment and recombination’ setting to identify any reassortment between the gene segments of Indian BTV strains. As evidence for reassortment, we took events detected by at least one of seven different recombination detection methods implemented in RDP: RDP, MAXCHI, and GENECONV methods in primary scanning mode and the Bootscan, CHIMAERA, SisScan, and 3SEQ methods in secondary scanning mode, each with a Bonferroni-corrected *P*-value cut-off of 0.05. Distribution of inferred breakpoints of events detected by at least five methods was also analysed.

Finally, linkage disequilibrium measures between pairs of single-nucleotide polymorphisms (SNPs) (within and among segments) were obtained using the D’ statistic implemented in DnaSP v6 (Librado and Rozas 2009).

### 2.8 Selection patterns

We used three complementary approaches to examine the BTV genome data for evidence of selection.

First, data were subjected to a neutrality test using Tajima’s *D* (Tajima 1989) with MEGA v7.0 (Kumar, Stecher, and Tamura 2016). Tajima’s *D* statistic measures the difference between two statistics describing the diversity within the alignment *(θ_π_* based on the average number of pairwise differences among sequences and *θ_S_* based on the number of segregating sites), standardised by the variance of this difference. Under the neutral evolutionary model, which assumes that polymorphisms segregate at mutation–drift equilibrium, Tajima’s *D* is expected to be null. Positive Tajima's *D* values indicate that there is an excess of polymorphisms at intermediate frequency and is typically associated with diversifying selection. Conversely, negative Tajima’s *D* values can be the result of purifying selection or a selective sweep (a beneficial allele has recently reached fixation due to strong positive selection), which both result in an excess of weakly divergent alleles. However, both positive and negative Tajima’s *D* values can also result from demographical and epidemiological processes.

Second, we tested for selection acting on particular amino acid sites by comparing the rates of non-synonymous (*d_N_*) and synonymous substitutions (*d_S_*), where *d_N_/d_S_* ratios are described by the parameter *ω*. Values of *ω* > 1 indicate positive selection, whereas a *ω *< 1 indicates the operation of purifying selection (Yang and Bielawski 2000). Analysis was performed with the program CODEML, which is part of the PAML package (Yang 1997). Likelihood ratio tests were computed to compare pairs of models: M0 (single ratio) with M3 (discrete), M1a (nearly neutral) with M2a (positive selection), and M7 (beta distribution) with M8 (beta distribution + positive selection) (Wong et al. 2004; Yang, Wong, and Nielsen 2005). The M0–M3 comparison is used as a test of variable *ω* among sites and M1a–M2a and M7–M8 comparisons are used as tests of positive selection (Yang 1997).

Finally, selection pressures were quantified for all codon alignments for each segment in a maximum likelihood framework. To determine the degree of natural selection acting on all protein coding regions, the average *d_N_/d_S_* ratio was estimated for the entire tree by using single likelihood ancestor counting (SLAC) (Kosakovsky Pond and Frost 2005). Positively selected codons along internal branches were detected with the fixed-effects likelihood (FEL) method (Kosakovsky Pond et al. 2006). Codons subject to episodic diversifying selection were identified with the mixed-effects model of evolution (MEME) method (Kosakovsky Pond et al. 2011), using a significance threshold of *P* ≤ 0.05. Fast unconstrained Bayesian approximation (FUBAR) was used to rapidly detect negative and positive selection by Bayesian MCMC analyses to robustly account for parameter estimation errors (Murrell et al. 2013). Codons with posterior probabilities ≥0.9 were reported as being either negatively or positively selected. All methods were implemented in the HyPhy package (Kosakovsky Pond, Frost, and Muse 2005), combined with the best-fit model of nucleotide substitution based on Akaike information criterion. All these analyses were accessed through the Datamonkey webserver (Delport et al. 2010).

## 3. Results

### 3.1 TMRCAs and evolutionary rates

Regression of root-to-tip genetic distance against sampling time (Supplementary Fig. S2) exposed sufficient temporal signal in each dataset (segment) to proceed with phylogenetic molecular clock analyses. BEAST (Drummond et al. 2012) estimates of the coefficients of variation (CoV) were consistently >2 for each BTV segment (Table 1), indicating that strict clock-like evolution could be rejected and validating our choice to use a relaxed molecular clock. However, due to this high degree of clock rate variability seen, we examined whether TMRCA estimates were affected by this. Exploratory analyses revealed that three isolates of BTV-10 (IND2014/BTV1_2014-12-06, IND2014/BTV2_2014-12-06, and BTV10IND2003K3_2003-08-01) in particular contributed to the high CoVs observed (MCC trees available at doi: 10.6084/m9.figshare.8275424). These three genomes exhibited >99 per cent similarity to an attenuated BTV strain, which is part of a live vaccine and thus might have been introduced into India through vaccinated animals or unauthorised use of American BTV-vaccines in the country (Maan et al. 2015). Repeating the BEAST analysis after excluding the BTV-10 sequences brought the CoV for the clock rate down for five of the ten segments but this had a negligible effect on their TRMCA estimates (Supplementary Table S3). For these segments, temporal calibration of BTV phylogenies was therefore not sensitive to clock rate variation. For the remaining segments, TMRCAs should be treated with more caution. However, given that TMRCAs showed limited variation across all ten segments, we conclude that clock rate variation was unlikely to have caused biases in our temporal estimation.

**Table 1.** Rates of nucleotide substitution and root height of each segment of BTV.

	CoV^a^				Substitution rate (subs/site/year)				Estimated date of MRCA^b^			
	Mean	Median	95% HPD^c^ lower	95% HPD^c^ upper	Mean	Median	95% HPD^c^ lower	95% HPD^c^ upper	Mean	Median	95% HPD^c^ lower	95% HPD^c^ upper
Seg-1	3.17	3.13	2.37	4.12	1.14E-03	1.13E-03	7.99E-04	1.47E-03	1953	1954	1941	1961
Seg-2	4.60	4.59	3.98	5.26	1.46E-02	1.45E-02	1.06E-02	1.86E-02	1937	1938	1918	1954
Seg-3	4.43	4.31	2.64	6.26	1.34E-03	1.33E-03	9.55E-04	1.76E-03	1956	1957	1949	1961
Seg-4	3.04	2.96	2.04	4.21	1.28E-03	1.27E-03	9.09E-04	1.63E-03	1956	1957	1947	1961
Seg-5	3.88	3.82	2.64	5.05	1.13E-03	1.12E-03	8.50E-04	1.41E-03	1955	1955	1947	1961
Seg6	4.95	5.03	3.57	5.79	9.40E-03	9.27E-03	5.96E-03	1.31E-02	1946	1946	1927	1961
Seg-7	5.36	5.30	3.97	6.79	2.28E-03	2.25E-03	1.54E-03	3.11E-03	1954	1956	1944	1961
Seg-8	3.05	2.97	1.98	4.21	1.22E-03	1.21E-03	8.54E-04	1.63E-03	1956	1957	1949	1961
Seg-9	3.45	3.39	2.44	4.68	7.81E-04	7.77E-04	5.29E-04	1.05E-03	1950	1951	1934	1961
Seg-10	2.28	2.21	1.45	3.17	1.06E-03	1.04E-03	7.31E-04	1.41E-03	1957	1957	1950	1961

Parameters were estimated based on ninety-two time-stamped Indian isolates in program BEAST v1.8.4 (Drummond et al. 2012) under a relaxed molecular clock and a flexible skygrid tree prior. Date of MRCA was deduced from the estimated mean root height.

a Coefficient of variation.

b Most recent common ancestor.

c Highest posterior density.

For Seg-2 and Seg-6, the mean TMRCAs of the sampled Indian BTV isolates were estimated to have existed in the late 1930s or early 1940s. The mean TMRCAs for other segments placed their ancestry in the 1950s (Table 1). Also taking the uncertainty of these estimates into account, these results indicate that the present genetic diversity seen in Indian BTV isolates has arisen within the past century.

Indian BTV viruses exhibited variable rates of evolutionary change across segments, ranging from 7.81E-04 (Seg-9) to 1.46E-02 (Seg-2) nucleotide substitutions per site per year (subs/site/year) with Seg-2 and Seg-6 showing higher rates than other segments (Table 1).

### 3.2 Sequence clustering and reassortment

**Figure 1.**
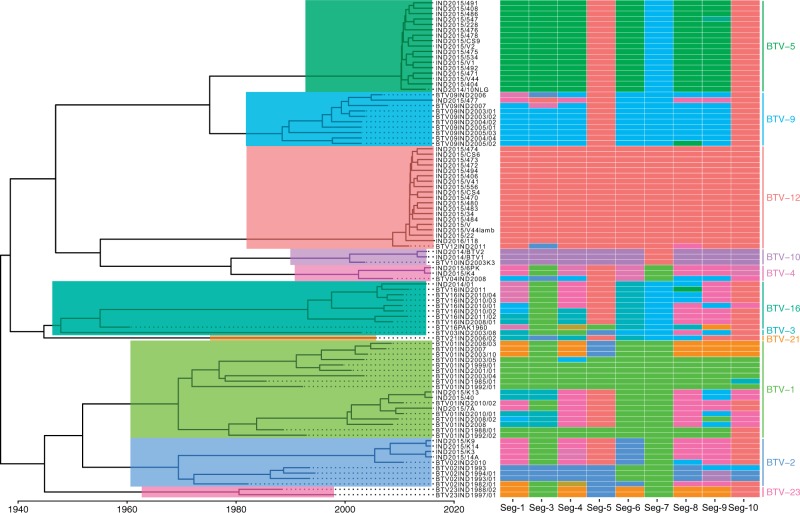
Phylogeny of Indian BTV Seg-2 and amino-acid based clusters of all genomic segments. Shown is a time-scaled maximum clade credibility (MCC) tree based on Seg-2 for ninety-two Indian BTV isolates, obtained using Bayesian phylogenetic inference in program BEAST (Drummond et al. 2012). Seg-2 sequences were assigned to amino-acid based clusters (Pessia et al. 2015), as highlighted on the tree, which corresponded almost perfectly to serotype (shown on the very right). The same clustering approach was used for all other segments in the genome, for which cluster memberships is shown as vignettes on the right side of the tree. Colours chosen to represent clusters are used consistently across all figures of the manuscript.

**Figure 2.**
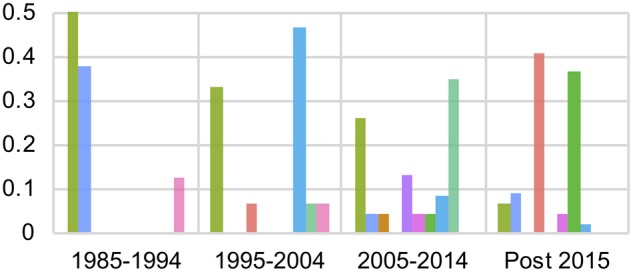
Seg-2 diversity in Indian BTV samples collected over four decades. The proportion of sequences falling into each of ten phylogenetic clusters for Seg-2 (largely equivalent to serotype, see Fig. 1) is shown for each decade: 1985–1994 (*n* = 8), 1995–2004 (*n* = 15), 2005–2014 (*n* = 23), 2015 and after (*n* = 44). Multiple Seg-2 types co-circulate within the same decade and dominant types vary between decades.

Time-scaled phylogenies and K-Pax2 clustering (Pessia et al. 2015) distinguished ten phylogenetic groups at the protein level for Seg-2 and nine groups for Seg-6 (Fig. 1, Supplementary Fig. S3). On Seg-2, K-Pax2 clustering was perfectly consistent with the serotype delineation (neighbour-joining), with the exception of one published BTV-3 sequence (BTV03/08) which grouped with BTV-16 sequences (Fig. 1). The serotypes dominating during each decade of sampling changed through time with Serotypes 1 and 9 being common early on whereas the most recent samples were almost exclusively comprised of Serotypes 5 and 12. During each decadal period, between three and eight clusters (i.e. serotypes) were co-circulating (Fig. 2).

**Figure 3.**
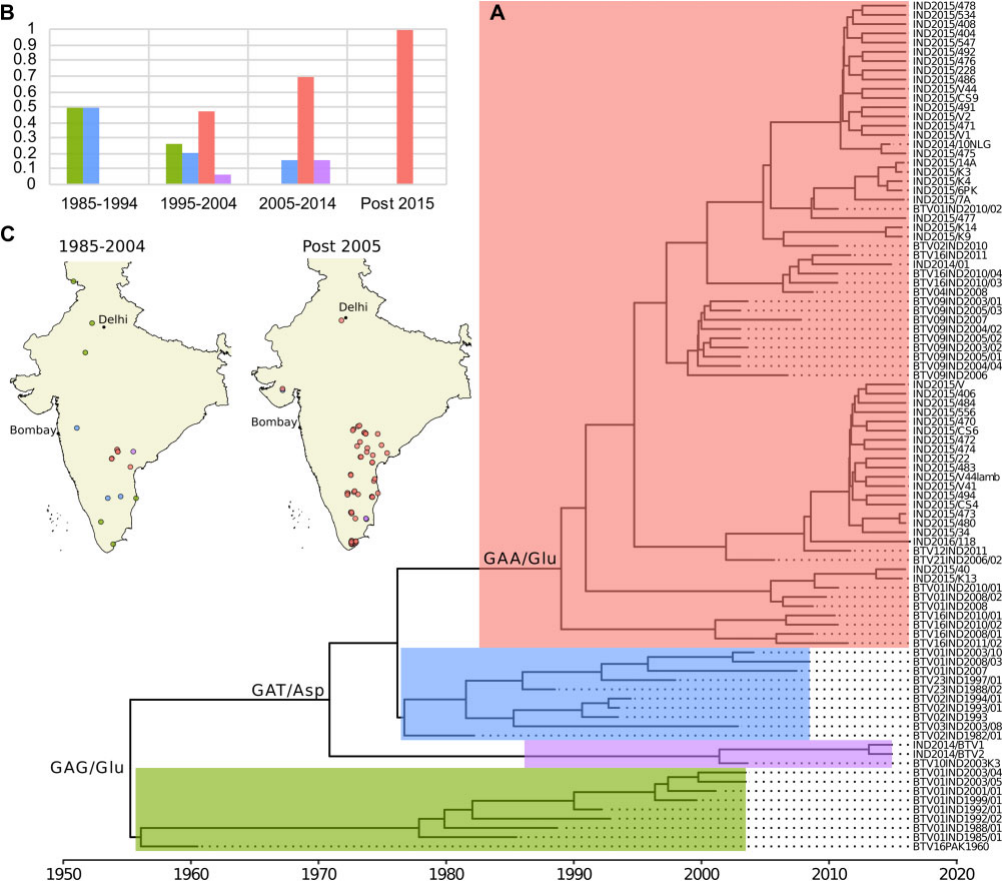
Changes in the phylogenetic diversity of Indian BTV Seg-5 sequences across time and space indicates that a single variant has risen to dominance in recent decades. (A) Seg-5 time-scaled MCC tree of the ninety-two Indian BTV isolates, which was obtained using Bayesian phylogenetic inference in program BEAST (Drummond et al. 2012). Sequences could be divided into four amino-acid based clusters (Pessia et al. 2015) represented by different colours on the tree. According to ancestral state reconstruction of Seg-5 amino acid sequences, the branch defining the largest cluster (shown in red) only includes a single unique non-synonymous change in codon 322 (Asp -> Glu). The inferred amino acid states in this position across other branches of the phylogeny are also shown. (B) Changes in the proportions of samples belonging to each of the four Seg-5 clusters over the past four decades, documenting the continuous rise in frequency of the red cluster, which has become the only variant found among samples collected since 2014. (C) The spatial distribution of Seg-5 clusters before and after 2005, confirming that the recent dominance of the red cluster applies across the Indian sub-continent.

For other segments, time-scaled phylogenies along with the K-Pax2 clustering revealed a high turnover rate involving on average the simultaneous circulation of 3.8 clusters per decade (Fig. 3, Supplementary Figs S4–S10). Notably, results for Seg-5 indicated a strong gradual reduction in the number of circulating variants through time (Fig. 3). The observed patterns suggest that a single variant has become completely dominant by 2015 and is associated with the full spectrum of genomic backgrounds found in Indian BTV strains currently circulating.

**Figure 4.**
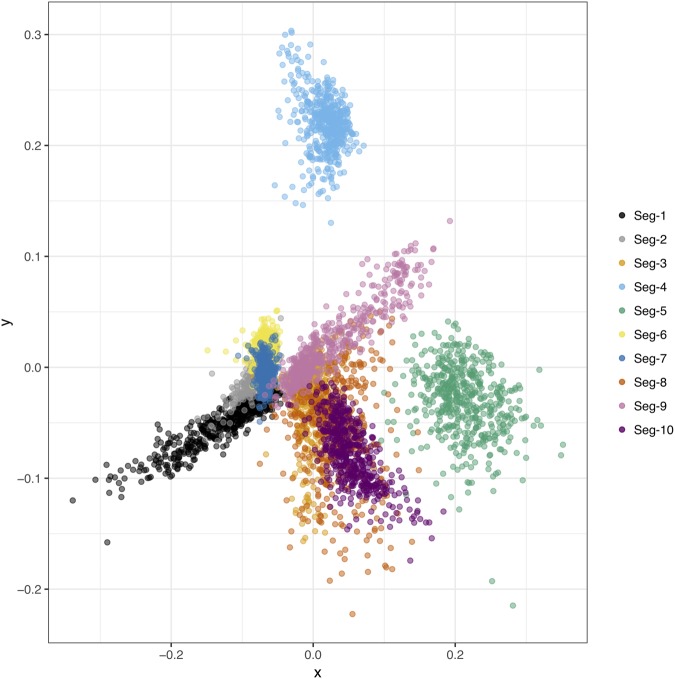
Multi-dimensional scaling (MDS) plot reflecting correlations in time to most recent common ancestor (TMRCA) between pairs of Indian BTV virus segments. In the absence of reassortment, segments are expected to exhibit highly correlated TMRCAs due to their evolutionary histories being tightly linked. MDS allows to depict the overall level of cross-correlation between all segments in two-dimensional space (Rambaut et al. 2008), where overlap between observations is indicative of shared evolutionary history (i.e. linkage) between segments. In contrast, segments broken up by reassortment are expected to occupy different regions in the plot. Temporal estimation was done separately for each of the ten BTV segments. Clouds of points reflect phylogenetic uncertainty based on 500 trees sampled in program BEAST (Drummond et al. 2012) for each segment, with pairwise comparisons to other segments being limited to viruses sampled in the same year. Only the first two dimensions of the scaling are shown.

We used MDS of TMRCAs (Bahl et al. 2013) to quantify the degree to which BTV segments had remained associated with each other through time and in the face of common genome reassortment. The analysis clearly indicated variable levels of reassortment for different segments (Fig. 4). Overlapping point clouds were seen for Seg-2, Seg-6, Seg-1, and Seg-7 as well as for Seg-3, Seg-8, Seg-9, and Seg-10, suggesting high level of linkage among segments within each of these groups. In contrast, Seg-4 and Seg-5 failed to show any clear association with other segments indicating that their evolutionary histories were more independent from those of other segments due to reassortment having disrupted these linkages.

**Figure 5.**
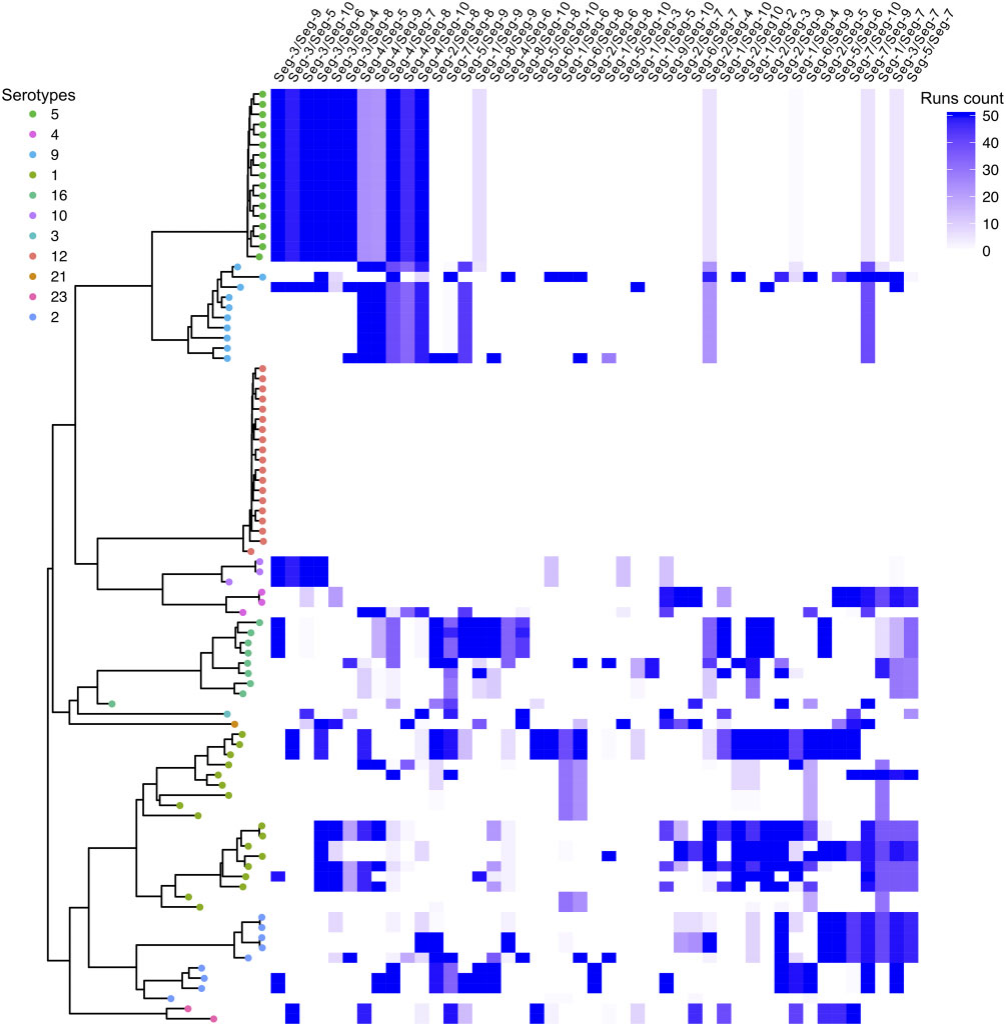
Tree topology inconsistencies between pairs of Indian BTV segments. Inconsistencies in tree topologies, indicative of genome reassortment, were identified using the program GiRaF (Nagarajan and Kingsford 2011). The number of independent runs (out of 50) with evidence of inconsistency is shown between each possible pair of segments (columns) for each BTV sample (rows). Samples are ordered according to the Seg-2 MCC tree (i.e. by serotype, Fig. 1). Segment pairs were ordered by reassortment pattern similarity using hclust, the Ward minimum variance clustering method, and Ward’s clustering criterion (Murtagh and Legendre 2014).

We also found a high number of well-supported incompatibilities between trees, indicating reassortment, using GiRaF (Fig. 5). This was seen in all ten segments, although the frequency of reassortment and the number of sequences impacted varied among them (Fig. 5, Supplementary Fig. S11). Tree topology inconsistencies were found for strains of all serotypes except BTV-12 which did not exhibit admixture at the amino-acid level either (Fig. 1).

Explicitly testing for intra-segment recombination and reassortment using RDP4, yielded no evidence of intracomponent recombination in any virus but strong evidence of reassortment. Indeed, we detected fifty-eight recombination/reassortment events by at least one of the seven methods used, forty-six being supported by five or more methods and inferred breakpoints always fell on or very close to concatenation sites between segments (Supplementary Fig. S12A). These results confirm that reassortment has been frequent in our data. Consistent with this conclusion, we found mean linkage disequilibrium between SNPs of the different segments to be low (Supplementary Fig. S12B).

### 3.3 Selection patterns

The population genetics analysis showed that, at both the nucleotide and the amino acid level, Seg-2, Seg-6, and Seg-7 presented positive Tajima’s *D* values which indicates an excess of polymorphisms at intermediate frequency and suggests positive diversifying selection is acting on these segments (Tajima 1989). In contrast, all other segments showed negative Tajima’s *D* values (Table 2) suggesting purifying selection and/or a recent population expansion. The single exception to this was Seg-9 which showed a Tajima’s *D* value close to zero.

**Table 2.** Population genetics analysis of the BTV isolates studied.

		*S*		*θ_S_*		*θπ*		*D*		PAML M3 parameter estimates						
	*N*	nuc	aa	nuc	aa	nuc	aa	nuc	aa	*ω*_0_	*ω*_1_	*ω*_2_	*p_0_*	*p_1_*	*p_2_*	*d_N_/d_S_*
Seg-1	92	1125	126	0.06	0.02	0.05	0.01	−0.32	−1.37	0.00	0.06	0.55	0.77	0.21	0.02	0.04
Seg-2	92	2496	854	0.17	0.17	0.42	0.50	5.29	6.64	0.01	0.04	0.15	0.25	0.51	0.24	0.13
Seg-3	92	749	40	0.05	0.01	0.04	0.00	−1.09	−1.96	0.00	0.03	NA	0.79	0.21	0.00	0.01
Seg-4	92	578	103	0.06	0.03	0.04	0.02	−1.06	−1.19	0.02	0.02	0.40	0.65	0.26	0.09	0.07
Seg-5	92	443	68	0.05	0.02	0.05	0.02	−0.47	−0.98	NA	0.02	0.33	0.00	0.91	0.09	0.06
Seg-6	92	928	269	0.11	0.10	0.28	0.22	4.82	4.04	0.00	0.01	0.07	0.35	0.49	0.16	0.05
Seg-7	92	337	20	0.06	0.01	0.14	0.02	4.01	2.18	0.00	0.00	0.03	0.27	0.58	0.15	0.01
Seg-8	92	319	73	0.06	0.04	0.03	0.02	−1.42	−1.40	0.01	0.39	7.59	0.83	0.17	0.00	0.11
Seg-9	93	370	135	0.07	0.08	0.08	0.08	0.25	0.01	0.08	0.08	0.81	0.40	0.29	0.31	0.31
Seg-9 NS4	93	57	11	0.05	0.03	0.04	0.01	−0.26	−1.58	0.03	NA	NA	1.00	0.00	0.00	0.08
Seg-10	92	172	25	0.05	0.02	0.03	0.01	−1.20	−2.02	0.00	0.25	0.25	0.88	0.11	0.01	0.05
Seg-10 NS5	92	29	24	0.03	0.08	0.02	0.05	−1.30	−1.02	0.00	9.98	73.49	0.27	0.70	0.03	6.56

*S*, *θ*_*S*_, *θ*_*p*_, and *D* statistics were computed in MEGA v7.0 (Kumar, Stecher, and Tamura 2016) at the nucleotide (nuc) and amino-acid (aa) levels. *ω*_0_, *ω*_1_, *ω*_2_ and *p_1_*, *p_2_* and *p_3_* are parameters estimated for the M3 model (discrete) which best fits data in PAML (Yang 1997). Mean *d_N_*/*d_S_* ratios were estimated using the single likelihood ancestor counting (SLAC) method (Kosakovsky Pond, Frost, and Muse 2005).

*N,* number of sequences; *S,* number of segregating sites; *D*, Tajima's D; *ω*, *d_N_*/*d_S_*; *p,* proportion of sites; NA, not applicable because *p* = 0.

No evidence for positive selection of amino acid replacements was found in the data with codeml (Yang 1997). While nine out of ten BTV segments supported the hypothesis of variable *ω* among sites with significant better support for M3 compare to M0, a high proportion of sites was inferred to correspond to *ω *< 1 (Table 2). The model M2a (positive selection) did not provide a significantly better fit for any segment than the nearly neutral model (M1a) which assumes a combination of purifying selection and neutral sites (*P*-values > 0.9). Similarly, the model M8 did not provide better fit than M7 (*P*-values > 0.09) (Supplementary Table S4). Further support for the BTV genomic segments being predominantly subject to purifying selection came from the mean *d_N_/d_S_* ratios, which ranged between 0.01 and 0.31 across the ten ORF (Table 2). Notably, Seg-10 alternate ORF, NS5, showed a higher *d_N_/d_S_* ratio of 6.56 (Table 2).

Positively and negatively selected codons were estimated by the FUBAR, FEL, and MEME methods of the HyPhy suit (Kosakovsky Pond, Frost, and Muse 2005). Only a single site on Seg-9 was inferred to be under positive selection by the FUBAR method (Table 3). FEL detected three more positively selected codons on Seg-2. MEME, which identifies fixed amino acid substitutions as well as codons under more sporadic positive selection, found a higher number of sites compared to other methods but these numbers remained low: six codons for Seg-1 and Seg-9, five for Seg-4, four for Seg-2, and one or two for the remaining segments (Table 3). Consistent with previous results, all segments contained an abundance of negatively selected codons ranging from 23 to 92 per cent for the FUBAR method and from 5 to 85 per cent for the FEL method. The alternative ORF for NS5 did not show evidence of negatively selected codons.

**Table 3.** Detection of positively and negatively selected codons.

		FUBAR^a^			FEL^b^			MEME^b^
	Total number of codons in the alignments	Number of sites under positive/ diversifying selection	Number and percentage of sites under negative/ purifying selection		Number of sites under positive/ diversifying selection	Number and percentage of sites under negative/ purifying selection		Number of sites under episodic positive/diversifying selection
Seg-1	1,302	0	936	71.89%	0	489	37.56%	6
Seg-2	978	0	816	83.44%	3	689	70.45%	4
Seg-3	901	0	689	76.47%	0	389	43.17%	2
Seg-4	644	0	390	60.56%	0	175	27.17%	5
Seg-5	552	0	307	55.62%	0	134	24.28%	1
Seg-6	528	0	487	92.23%	0	448	84.85%	1
Seg-7	349	0	304	87.11%	0	219	62.75%	1
Seg-8	354	0	155	43.79%	0	76	21.47%	2
Seg-9	330	1	76	23.03%	1	70	21.21%	6
Seg-9 NS4	77	0	13	16.88%	0	1	1.30%	0
Seg-10	229	0	83	36.24%	0	12	5.24%	0
Seg-10 NS5	59	8	0	0.00%	7	0	0.00%	1

Positively and negatively selected codons were estimated by the FUBAR, FEL, and MEME methods of the HyPhy suit (Kosakovsky Pond, Frost, and Muse 2005).

a Codons with posterior probabilities >0.9 are reported.

b Codons with *P*-values of <0.05 are reported.

Motivated by the striking observation that a single variant of Seg-5 appeared to have replaced all other variants, we examined codon substitutions of the NS1 gene ORF (Seg-5) that could be responsible for conferring higher viral fitness to the dominant variant using DnaSP v6 (Librado and Rozas 2009). We were able to identify a single non-synonymous change at the tree node delineating the emerging cluster which involved the replacement of an aspartic acid by a glutamic acid at codon position 322 (nucleotide position 966) (Fig. 3). This codon was not among the sites inferred to be under either positive of negative selection in our previous analysis (data not shown).

## 4. Discussion

Previous studies based on field isolates of BTV have shown that reassortment is common where multiple distinct strains of the virus circulate and/or live attenuated vaccines have been used (Heidner et al. 1991; Nomikou et al. 2015; Schirtzinger et al. 2018). Our findings confirm that genetic exchange by reassortment has had a major impact on the genomic composition of Indian BTV strains. The current work further indicates that many of these reassortment events must have occurred in recent decades given that our molecular clock estimates for all ten segments point to most recent common ancestors of Indian isolates dating to the middle of the 20th century, coinciding with dates of the first reports of BT disease on the Indian sub-continent in the 1950s and 1960s (Sarwar 1962; Sapre 1964). This suggests that most BTV strains currently circulating in India might have been introduced during that period. However, we cannot exclude the possibility that other strains had been present previously but that their diversity during that time was reduced to a single lineage or that these earlier strains might have since become extinct (Holmes 2003), resulting in an underestimate of the real origin of segments.

Examining the correlation among ages of most recent common ancestors using MDS suggests that two core sets of segments (Seg-1, Seg-2, Seg-6, and Seg-7 on one hand and Seg-3, Seg-9, and Seg-10 on the other hand) are less permissive to be broken up by reassortment than a third set comprising Seg-4 and Seg-5. These groups of associated segments inferred for Indian BTV virus segments are noticeably different from what was observed for BTV in Europe (Nomikou et al. 2015) where Seg-1, Seg-3, Seg-4, Seg-5, Seg-8, and Seg-9 grouped together while Seg-7 and Seg-10 failed to show any clear association with other segments and Seg-2 and Seg-6 showed weak evidence for a connection. Whether these differences are due to biological reasons or chance will require further investigation.

While reassortment in segmented-RNA viruses appears to often be a selectively neutral process (Marshall et al. 2013; Shaw et al. 2013), a specific combination of viral segments might quickly rise to dominance if it results in a fitness advantage for the virus (McDonald and Patton 2011; Shaw et al. 2013; Anbalagan et al. 2014; Lu, Lycett, and Leigh Brown 2014; Lago et al. 2017). Our results show that a particular BTV Seg-5 variant has strongly increased in frequency over the past decades, consistent with this kind of selective sweep scenario. We found Indian BTV strains to have highly admixed genomes and our MDS plot analysis shows that Seg-5 does not share its evolutionary history with any other segment. A previous study had suggested that the now dominant variant of Seg-5 was introduced into India as part of an exotic western virus strain or combination of strains (Maan et al. 2015). It is therefore plausible that frequent reassortment events have allowed the successful Seg-5 variant to become associated with a large spectrum of genomic backgrounds in India. Indeed, all but three of the sixty-three BTV viruses collected after 2008 contained this variant (Fig. 3). Unexpectedly, the number of tree incompatibilities detected (and thus the number of reassortment events) involving Seg-5 is quite low compared to other segments (Fig. 5, Supplementary Fig. S11). One possible explanation is that the proposed takeover by the dominant variant occurred very rapidly and, by replacing all other strains, has limited the opportunity to detect any further reassortment events involving Seg-5.

While a genomic variant can also increase in a population as a consequence of positive selection, we found no indication for this in Seg-5. Instead, the observed increase in nucleotide diversity in this segment over time appears to be due to a population expansion following a bottleneck. Indeed, to understand the role of positive selection on evolution of Seg-5 of Indian BTV strains, we analysed sequences using a suite of methods (FUBAR, SLAC, and FEL), all of which failed to identify any positively selected codons. The MEME method identified only one site with evidence of episodic diversifying selection (codon 521). In contrast, most codons were identified to be under negative/purifying selection. This indicates strong structural and functional constraints for NS1 to evolve and suggests that the putative fitness benefits conferred by the invading Seg-5 variant applied regardless of the genotypic makeup of the other segments and did not require any further mutational changes of NS1. This supports the hypothesis that the invasive variant of Seg-5 underwent a recent selective sweep based on pre-existing genotypic and phenotypic characteristics.

The Seg-5 phylogeny of Indian BTV isolates revealed a single non-synonymous change in the emerging variant at the internal node corresponding to the basal branch of the cluster (codon 322, amino acid change: aspartic acid -> glutamic acid, Fig. 3). Since this replacement is the only one that separates this variant from the other Seg-5 variants it has replaced, one might expect that this change must be responsible for the apparent selective advantage. However, more basal branches in the Seg-5 phylogeny and some of the older sequences of BTV-1 and BTV-16 also harboured a nucleotide sequence coding for a glutamic acid at this codon position (green cluster in Fig. 3). At least two non-exclusive hypotheses might explain this. First, the selective environment in which BTV circulates could have changed during the last two decades (e.g. host immune status changes, increase of the viral diversity, or modification of the vector distribution and activity patterns) and a specific amino-acid could have become advantageous in this new environment when it had not been before. Secondly, it is possible that the variant success observed in recent years might be due to epistasis, i.e. to specific interactions with concurrent substitutions in other BTV proteins. Epistatic effects play a central role in shaping the path of viral evolution in fitness landscapes of RNA viruses (Michalakis and Roze 2004; Sanjuan, Moya, and Elena 2004; Elena, Solé, and Sardanyés 2010) but are generally difficult to establish. Further *in vitro* experiments are required to elucidate the specific mechanisms underlying the differences in fitness and selection across Indian BTV genomes and high-throughput genetics approaches could be used to identify potentially relevant epistatic interactions involved (Wu et al. 2016).

An earlier study had reported that the estimated substitution rates for global isolates of BTV were comparable across segments, except for an order of magnitude lower rate for Seg-6 (5.22E-5) (Carpi, Holmes, and Kitchen 2010). Our estimates for BTV in India indicate that genes encoding for proteins interacting with host immunity (outer capsid) on Seg-2 and Seg-6 evolve at higher mean rates (1.47E-2 and 9.36E-3, respectively) and have longer TMRCA’s than genes on other segments (<2.25E-3, Table 1). Similar differences have been observed between outer and inner coat protein genes in the case of influenza viruses (Worobey, Han, and Rambaut 2014). Whereas variants of outer capsid proteins will be maintained by balancing selection, those of inner capsid and non-structural genes are likely to be replaced continuously by new introduced variants through serial hitchhiking. This might result in an allele changing in frequency not because it is under natural selection itself, but because it is associated with another gene that is undergoing a selective sweep- and genetic drift.

Deviation from neutrality tests also showed two contrasting patterns; Seg-2, Seg-6 as well as Seg-7 (which encodes for the intermediate capsid protein VP7) displayed positive *D* values, whereas other segments displayed negative *D* values or values close to 0. Combined with other selection detection methods, our results provide evidence for differences in the predominant mechanisms of evolution of these genes. Outer capsid protein genes may be subject to positive and balancing selection leading to the generation of diverse serotypes and their variants, whereas evolution of all other genes appears to be driven by a combination of neutral and purifying selection. This is consistent with previous studies where purifying selection was reported to be one of the major evolutionary mechanisms in viruses including BTV (e.g. Balasuriya et al. 2008; Worobey, Han, and Rambaut 2014).

In conclusion, our comprehensive analysis of BTV evolution in India, a global hotspot of BTV diversity, provides new insights into the role of reassortment in driving rapid evolutionary change in segmented viruses. Significantly, we present strong evidence for a selective sweep event that has allowed a single variant of NS1 to invade all BTV serotypes currently circulating in India through reassortment, though the basis for this apparent selective advantage remains yet unclear. The spread of this variant throughout the Indian BTV population may be related to the role of NS1 as a positive regulator of VP synthesis and as an antigen raising a protective T cell response (Boyce, Celma, and Roy 2012; Marín-López et al. 2018). This finding has important implications. It demonstrates the high potential for new BTV variants to spread across a large geographic area (Indian sub-continent) within a relatively short period of time (few decades). The same process could also facilitate the dissemination of novel phenotypes such a high virulence strains. This is particularly true in India where several incursions of new serotypes and variants have already been reported during the last decade (Maan et al. 2012a,b; Rao, Reddy, and Hegde 2015; Rao, Hegde, and Reddy 2016; Reddy et al. 2016; Hemadri et al. 2017). Specifically, it is conceivable that the recent evolutionary dynamics in Seg-5 we have identified are connected to the observation of more virulent BTV forms in India (Prasad et al. 2009; Chand et al. 2015; Rao et al. 2016). Our work has thus laid the groundwork for future studies that should focus on characterising the mechanisms underlying differences in fitness and selection across the BTV genome.

## Ethics

This research project did not require any ethical approval.

## Supplementary Material

vez027_Supplementary_Data
