# Supplementary material for: Contrasting selective patterns across the segmented genome of bluetongue virus in a global reassortment hotspot
Source: Virus Evol. 2019 Aug 5;5(2):vez027. doi: 10.1093/ve/vez027 (PMC6680063; doi:10.1093/ve/vez027)
Supplement: vez027_Supplementary_Data [file vez027_supplementary_data.zip › SupplementaryData_Legends.docx]

**Contrasting selective patterns across the segmented genome of bluetongue virus in a global reassortment hotspot**

**SUPPLEMENTARY DATA - Legends**

**Table S1: Genomes used in this study and associated metadata.**

**Table S2: Mapping and coverage statistics of the BTV samples sequenced at MRC-CVR, UK.** The number of mapped reads, the breadth and the percentage of sites on the genome with coverage was calculated across for the whole genome and by segment for each BTV sample sequenced at MRC-CVR, UK.

**Table S3: Rates of nucleotide substitution and root height of each segment of BTV.** Parameters were estimated based on the 92 time-stamped Indian isolates as well as with serotype 10 isolates excluded (IND2014/BTV1_2014-12-06, IND2014/BTV2_2014-12-06, BTV10IND2003K3_2003-08-01) in program BEAST v1.8.4 [55] under a relaxed molecular clock and a flexible skygrid tree prior. Date of MRCA were deducted from the estimated mean root height.

**Table S4: PAML selection analysis.** For all segments, shown is the log likelihood of each PAML models and likelihood ratio tests. Nine out of ten BTV segments supported the hypothesis of variable *ω* among sites with significant better support for M3 compare to M0.

**Figure S1: Spatial distribution of Indian BTV samples.**

Spatial distribution of samples from which genomes were analysed in this study. Approximate geo-references (latitude and longitude) were obtained using GoogleMaps for those cases where only rough meta-data on spatial origin were available (*e.g.* region or city).

**Figure S2: Clock-likeness of Indian BTV genomes.**

Regression of root-to-tip genetic distance against sampling time were computed using TempEst v1.5.1 [54] for each segment using two rooting methods (best fit in TempEst and midpoint). The three divergent BTV-10 sequences were removed from the analysis for all segments except Seg-2, Seg-6 and Seg-5 and sequences from Seg-7 and Seg-9 were separated into two clades to reduce the time period over which the extrapolation is made. Sufficient and robust temporal signal was observed in each dataset to proceed with phylogenetic molecular clock analyses.

**Figure S3: Phylogeny of BTV Seg-6 and amino-acid based clusters.**

Seg-6 time-scaled MCC tree of the 92 Indian BTV isolates which was obtained using Bayesian phylogenetic inference in program BEAST [55]. Seg-6 sequences were assigned to amino-acid based clusters [60], as highlighted on the tree. Proportions of samples belonging to each Seg-6 cluster across decades are reported in the chart on the top left.

**Figure S4: Phylogeny of BTV Seg-1 and amino-acid based clusters.**

Seg-1 time-scaled MCC tree of the 92 Indian BTV isolates. See Fig. S2 for further details.

**Figure S5: Phylogeny of BTV Seg-3 and amino-acid based clusters.**

Seg-3 time-scaled MCC tree of the 92 Indian BTV isolates. See Fig. S2 for further details.

**Figure S6: Phylogeny of BTV Seg-4 and amino-acid based clusters.**

Seg-4 time-scaled MCC tree of the 92 Indian BTV isolates. See Fig. S2 for further details.

**Figure S7: Phylogeny of BTV Seg-7 and amino-acid based clusters.**

Seg-7 time-scaled MCC tree of the 92 Indian BTV isolates. See Fig. S2 for further details.

**Figure S8: Phylogeny of BTV Seg-8 and amino-acid based clusters.**

Seg-8 time-scaled MCC tree of the 92 Indian BTV isolates. See Fig. S2 for further details.

**Figure S9: Phylogeny of BTV Seg-9 and amino-acid based clusters.**

Seg-9 time-scaled MCC tree of the 92 Indian BTV isolates. See Fig. S2 for further details.

**Figure S10: Phylogeny of BTV Seg-10 and amino-acid based clusters.**

Seg-10 time-scaled MCC tree of the 92 Indian BTV isolates. See Fig. S2 for further details.

**Figure S11: Distribution of phylogenetic tree inconsistencies across the BTV genome.**

Proportion of sequences for which evidence for phylogenetic tree inconsistency was very strong (supported by 50 out of 50 runs) as inferred using GiRaF [64] for each pair of segments.

**Figure S12: Detection of intrasegment recombination and reassortment.**

Recombination/reassortment occurrences were testing using RDP v4.97 [67] and SNPs linkage disequilibrium in DnaSP v6 [68]. A) Distribution of breakpoints identified by RDP for the 46 events being supported by at least 5 out of 7 of the methods used. Breakpoints are mostly distributed around the alignment concatenation sites (delimited by the red vertical lines) indicating an absence of intrasegment recombination and frequent reassortment in the data. B) Heatmap of mean linkage disequilibrium between SNPs of the different segments. Low intersegment linkage values support the assumption of frequent reassortment.
