# Supplementary figures and images for: Contrasting selective patterns across the segmented genome of bluetongue virus in a global reassortment hotspot
Source: Virus Evol. 2019 Aug 5;5(2):vez027. doi: 10.1093/ve/vez027 (PMC6680063; doi:10.1093/ve/vez027)

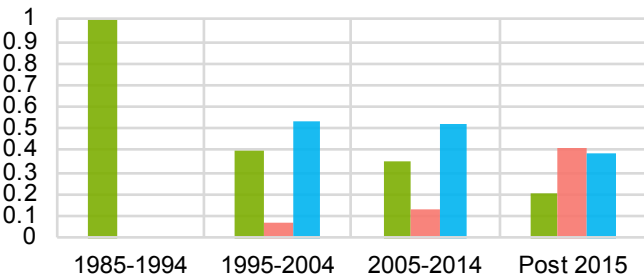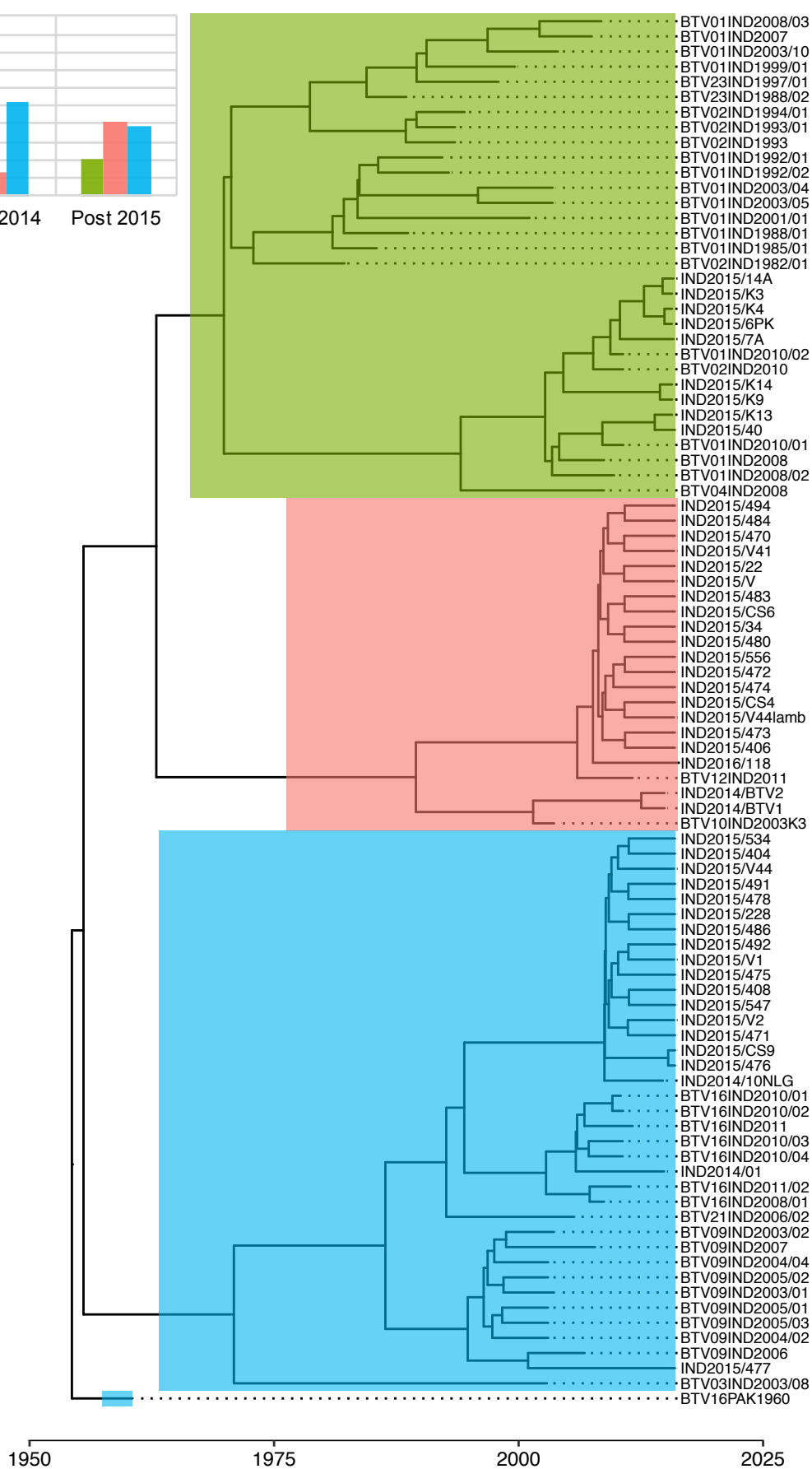

Supplement: vez027_Supplementary_Data [file vez027_supplementary_data.zip › FigureS7_Seg7_sub1.pdf]

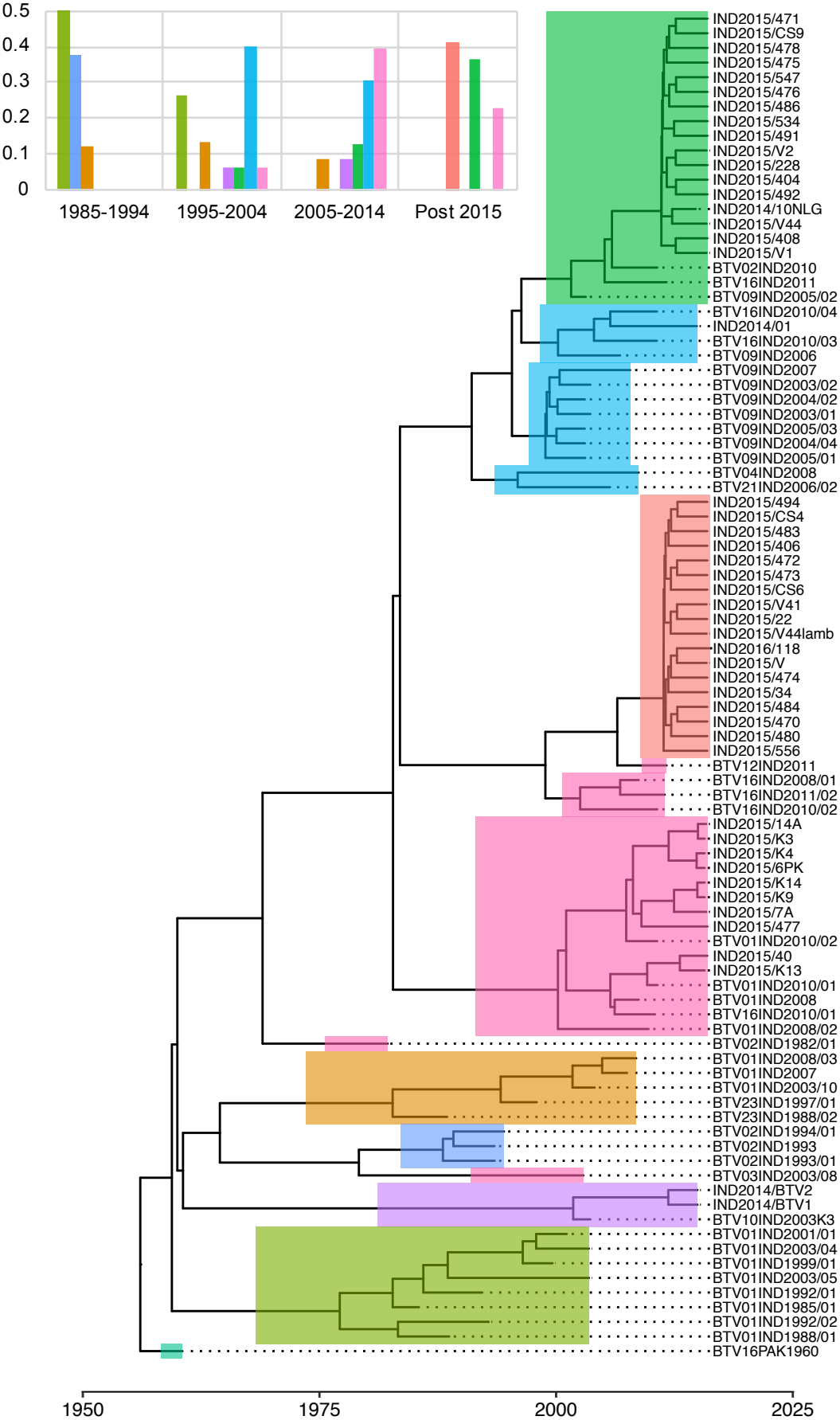

Supplement: vez027_Supplementary_Data [file vez027_supplementary_data.zip › FigureS8_Seg8_sub1.pdf]

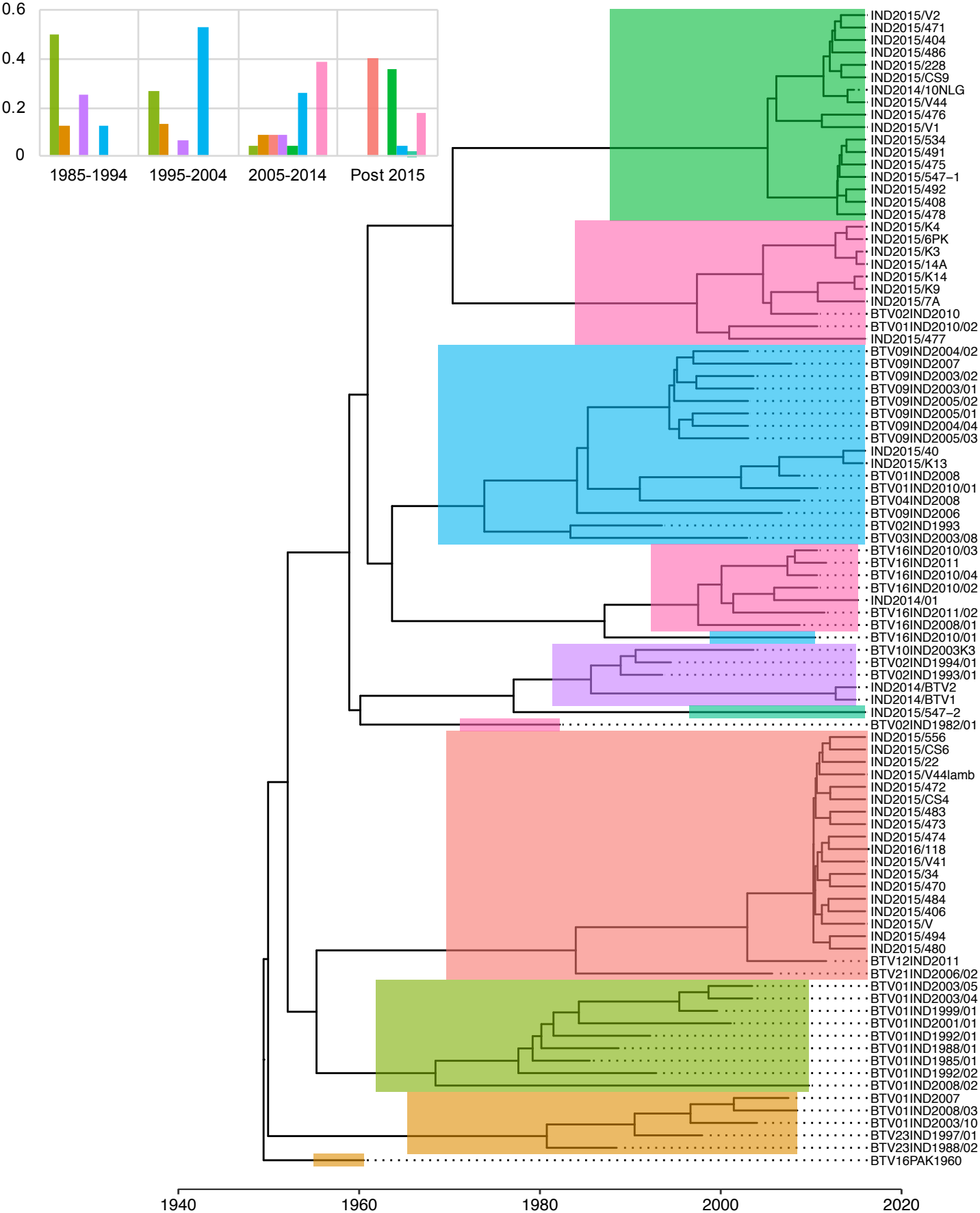

Supplement: vez027_Supplementary_Data [file vez027_supplementary_data.zip › FigureS9_Seg9_sub1.pdf]

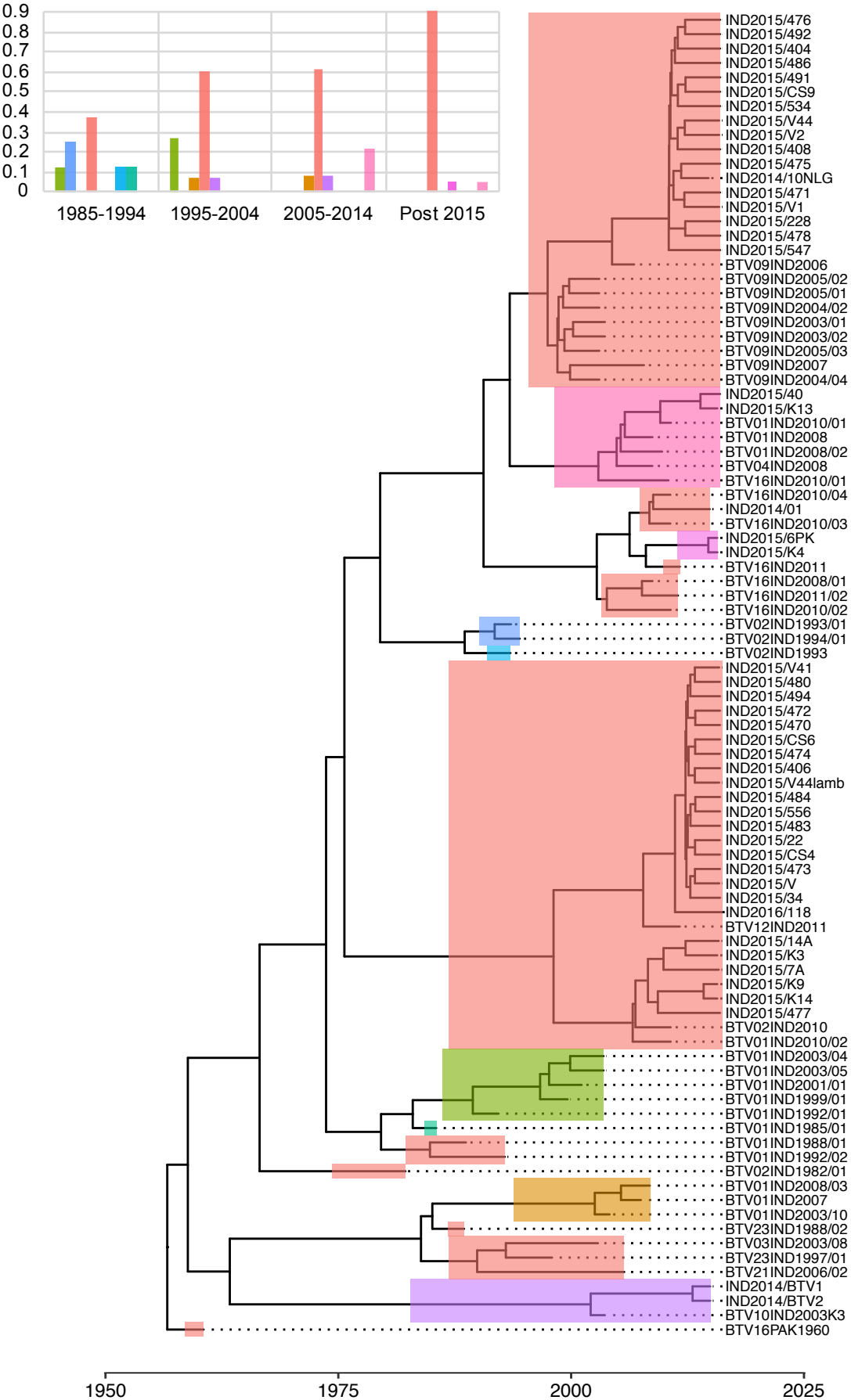

Supplement: vez027_Supplementary_Data [file vez027_supplementary_data.zip › FigureS10_Seg10_sub1.pdf]

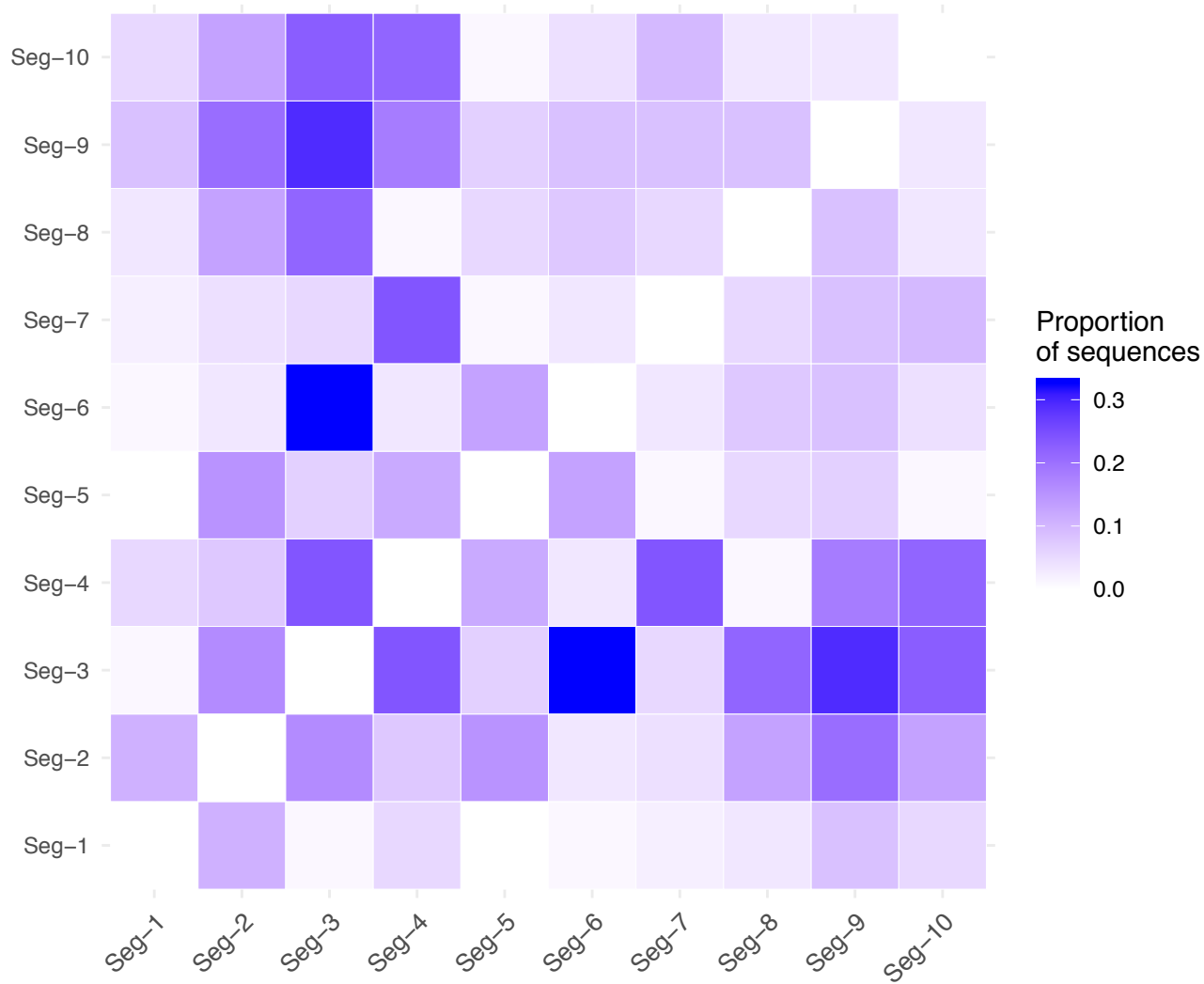

Supplement: vez027_Supplementary_Data [file vez027_supplementary_data.zip › FigureS11_rev1.pdf]

**A**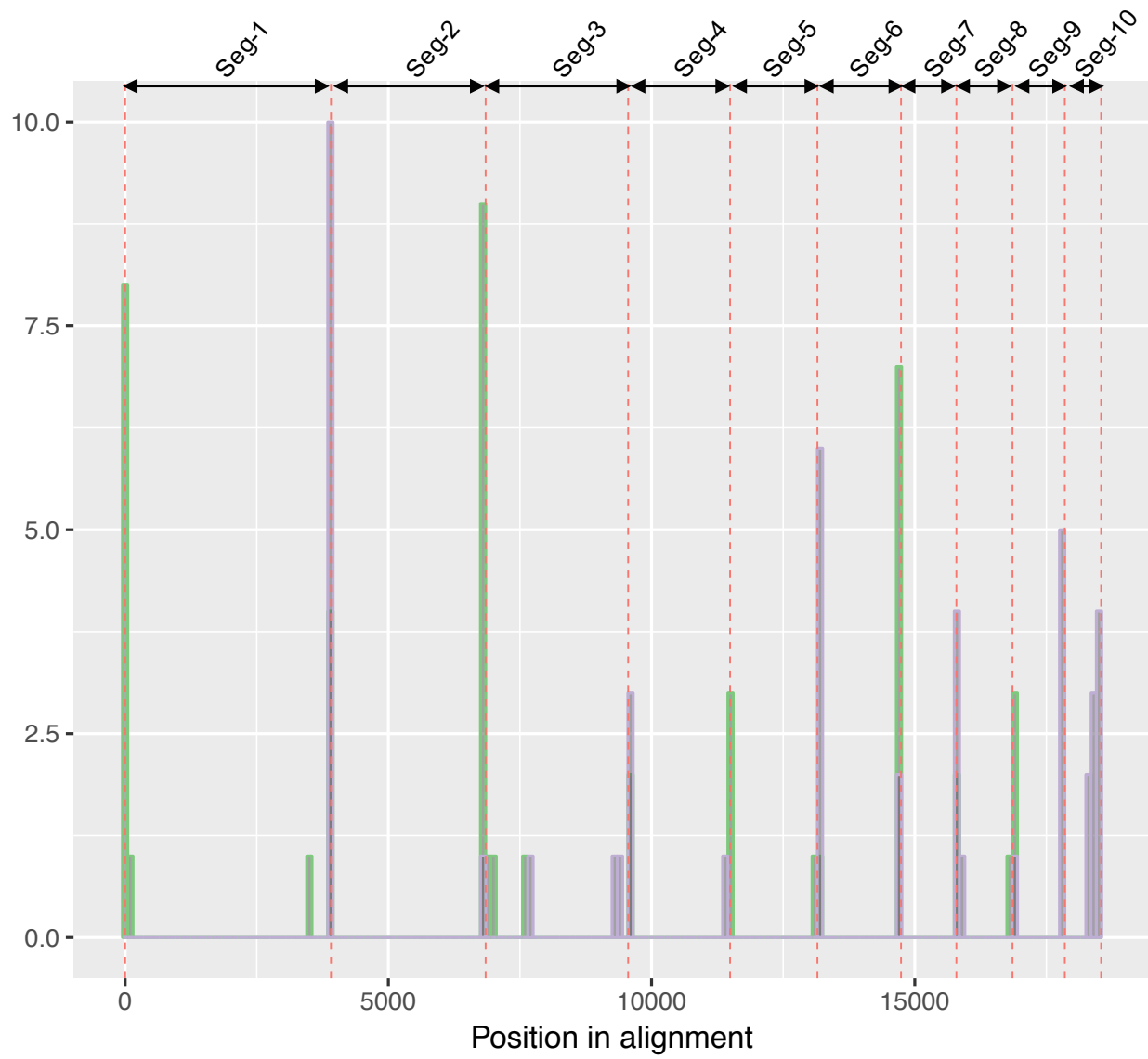**B**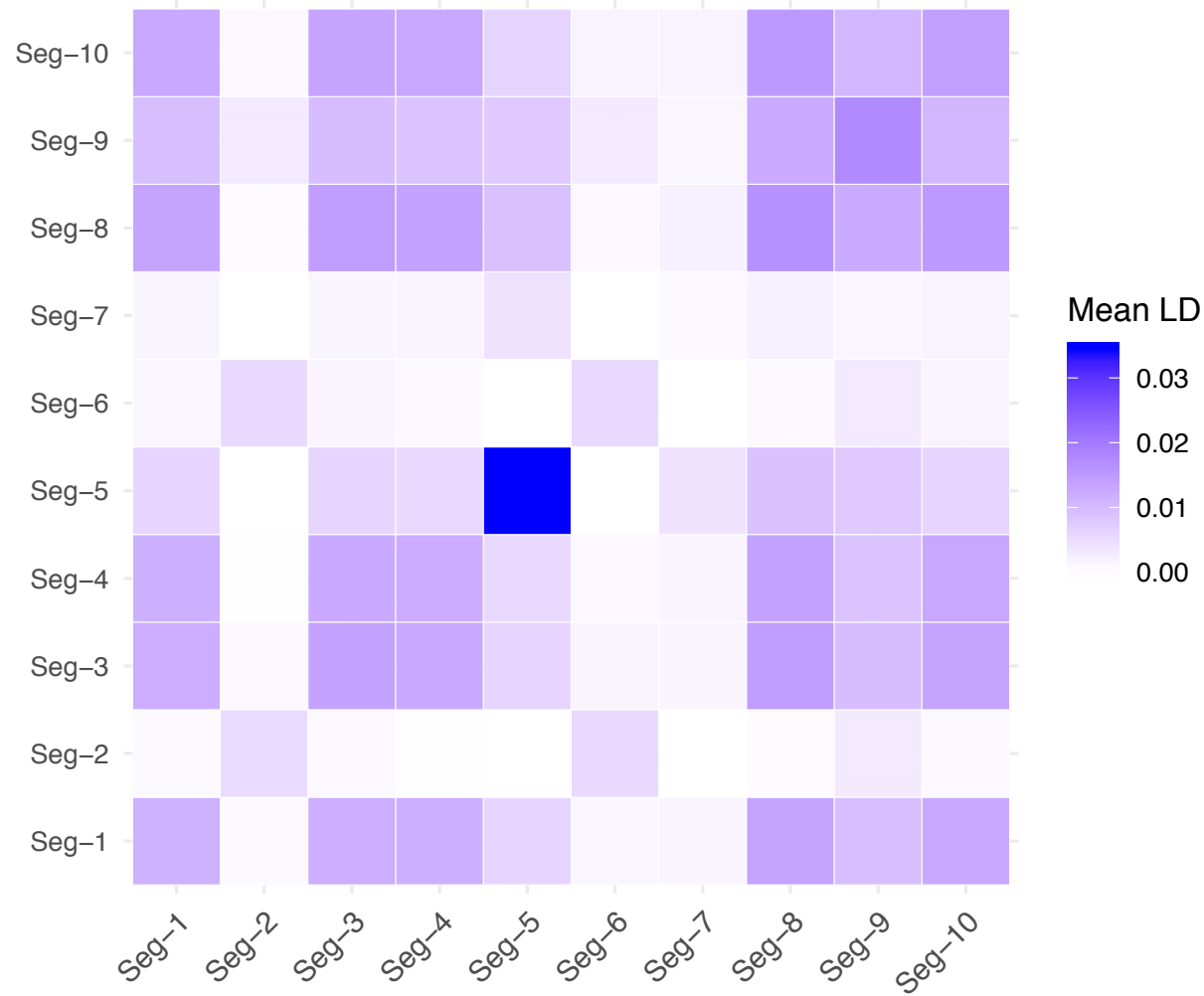

Supplement: vez027_Supplementary_Data [file vez027_supplementary_data.zip › FigureS12_RDPandD_rev1.pdf]

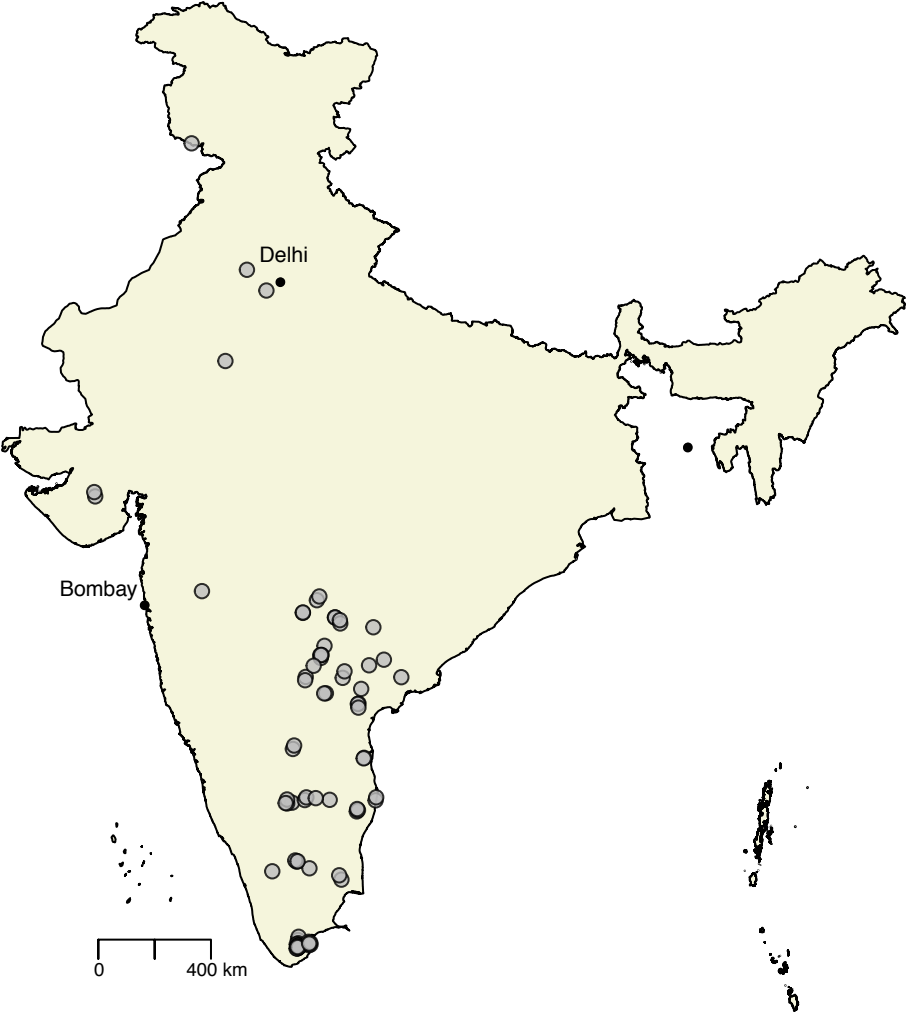

Supplement: vez027_Supplementary_Data [file vez027_supplementary_data.zip › FigureS1_SpatialDistribSamples_sub1.pdf]

Root-to-tip divergence

Seg-1

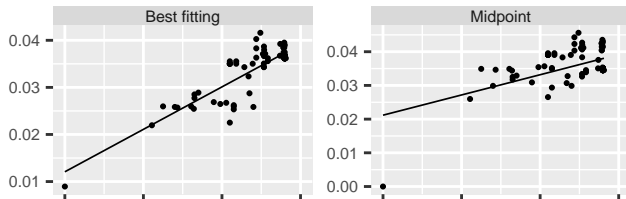

Seg-2

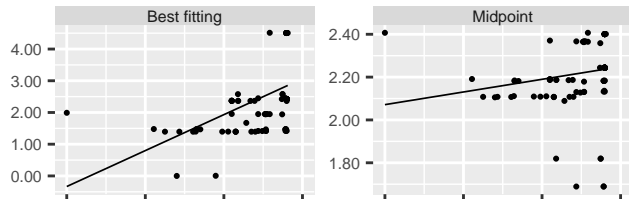

Seg-3

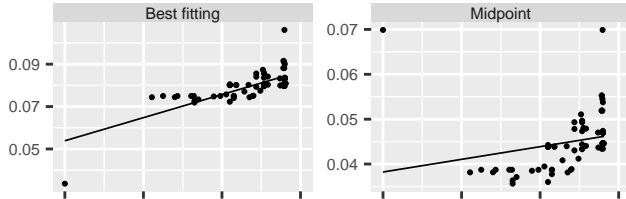

Seg-4

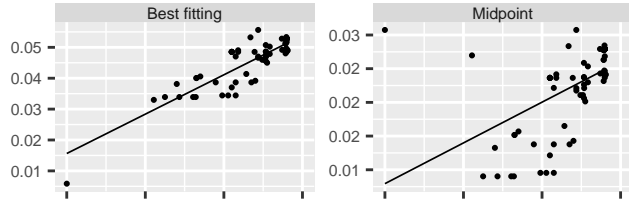

Seg-5

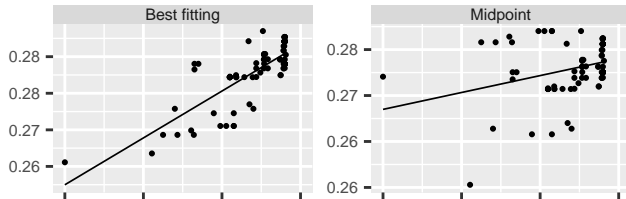

Seg-6

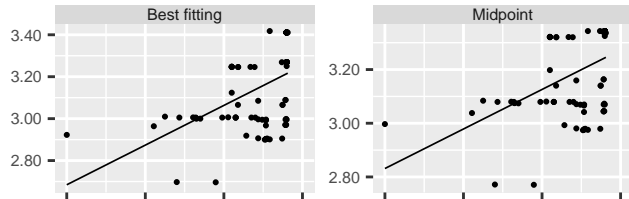

Seg-7

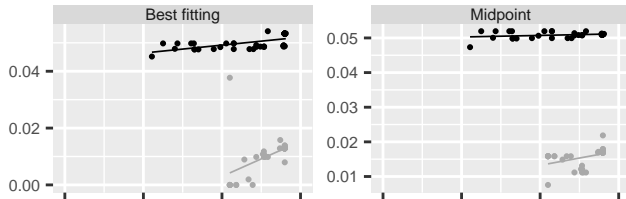

Seg-8

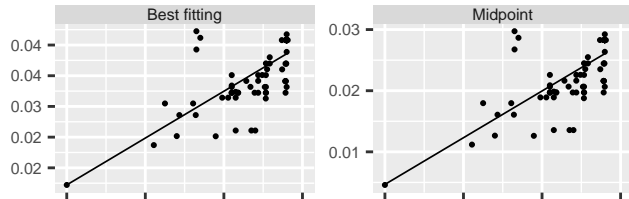

Seg-9

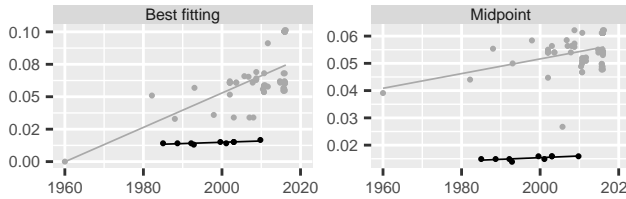

Seg-10

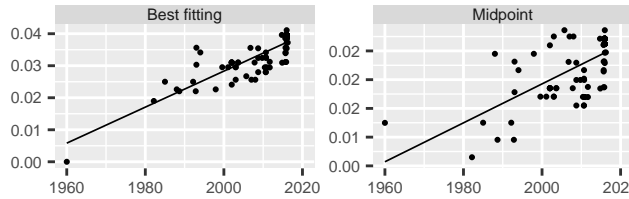

Date

Supplement: vez027_Supplementary_Data [file vez027_supplementary_data.zip › FigureS2_RTTD_plots_rev3.pdf]

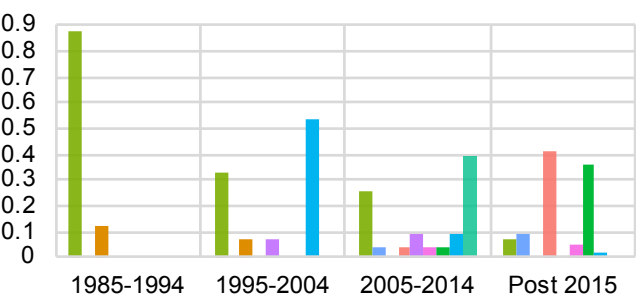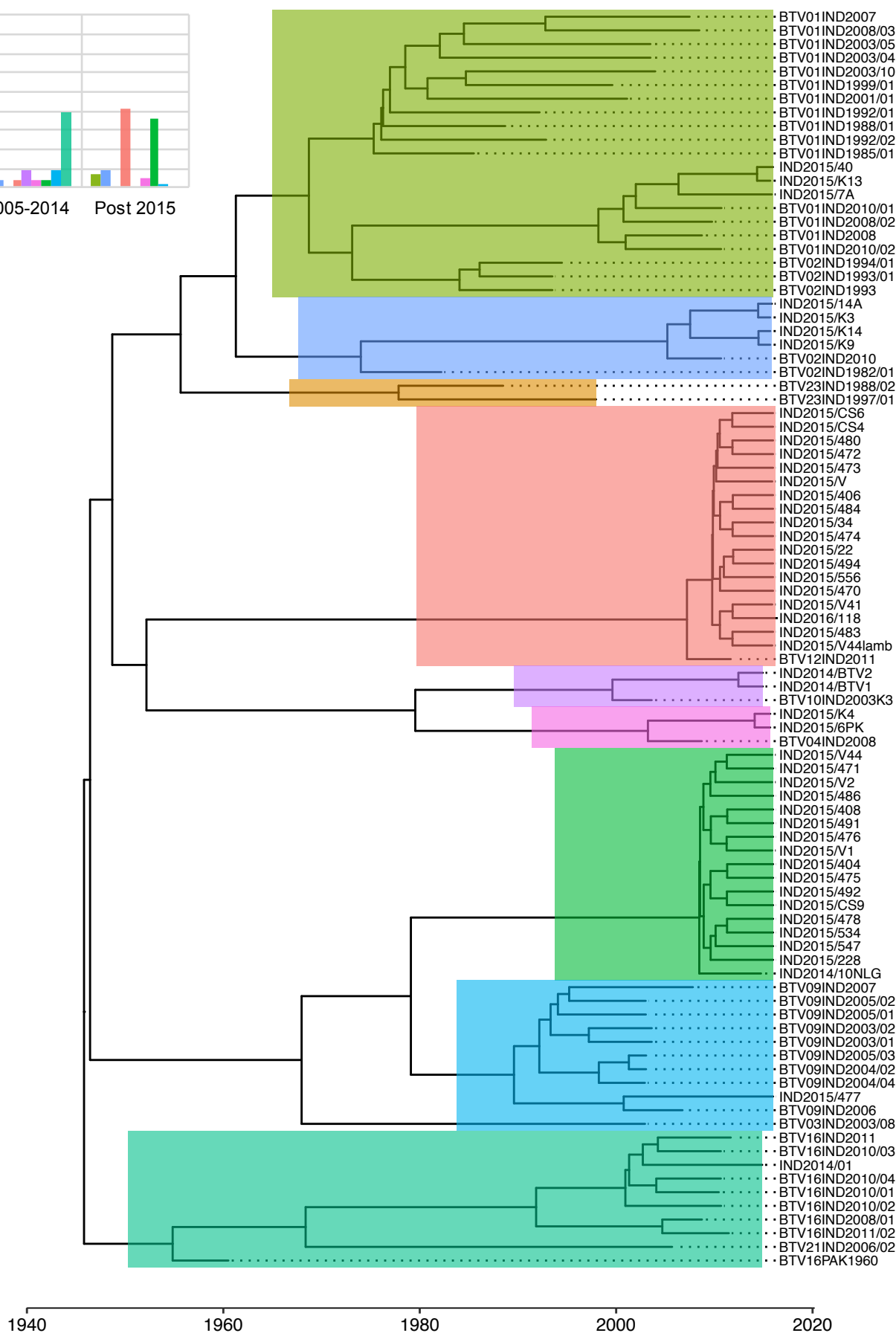

Supplement: vez027_Supplementary_Data [file vez027_supplementary_data.zip › FigureS3_Seg6_sub1.pdf]

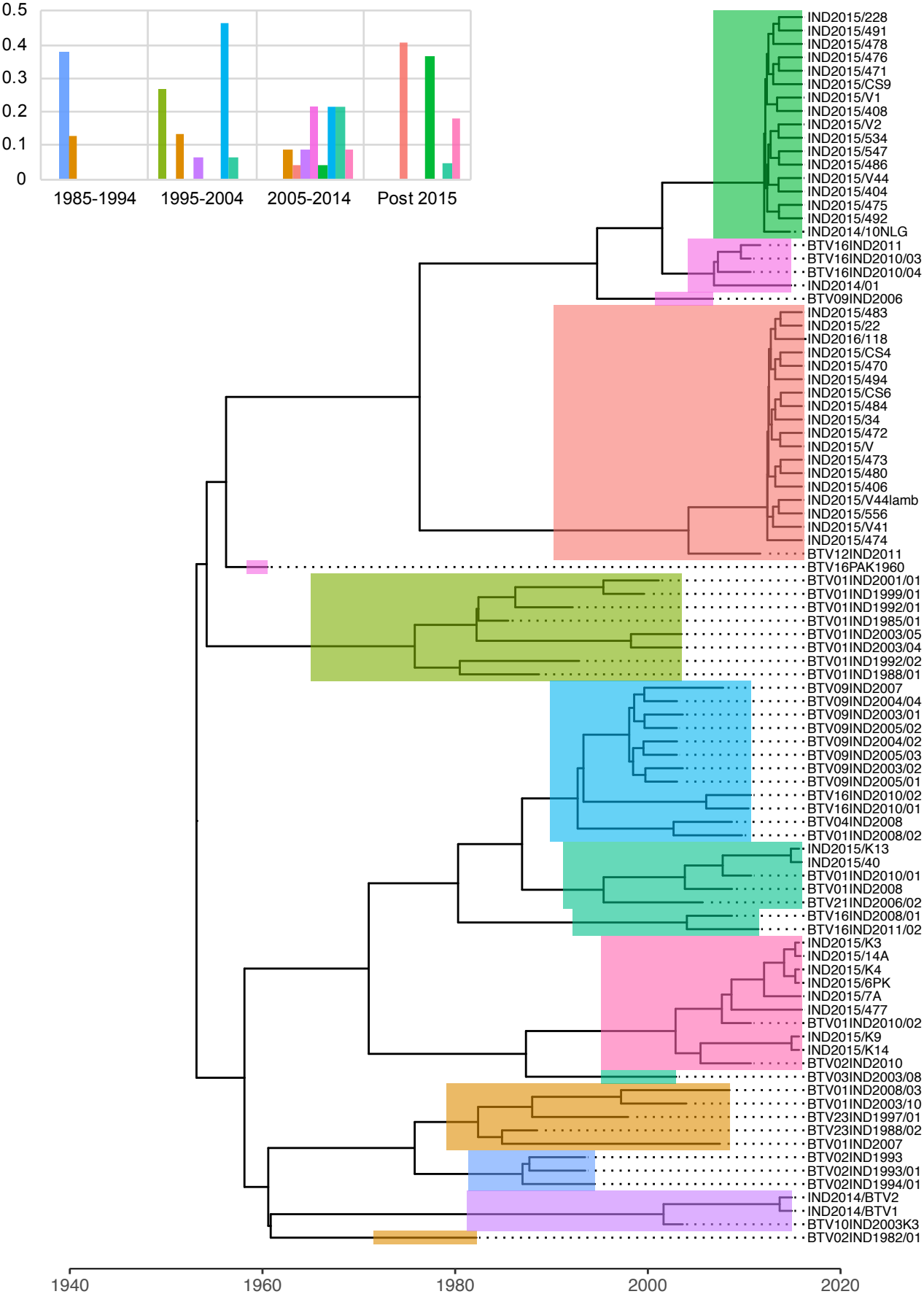

Supplement: vez027_Supplementary_Data [file vez027_supplementary_data.zip › FigureS4_Seg1_sub1.pdf]

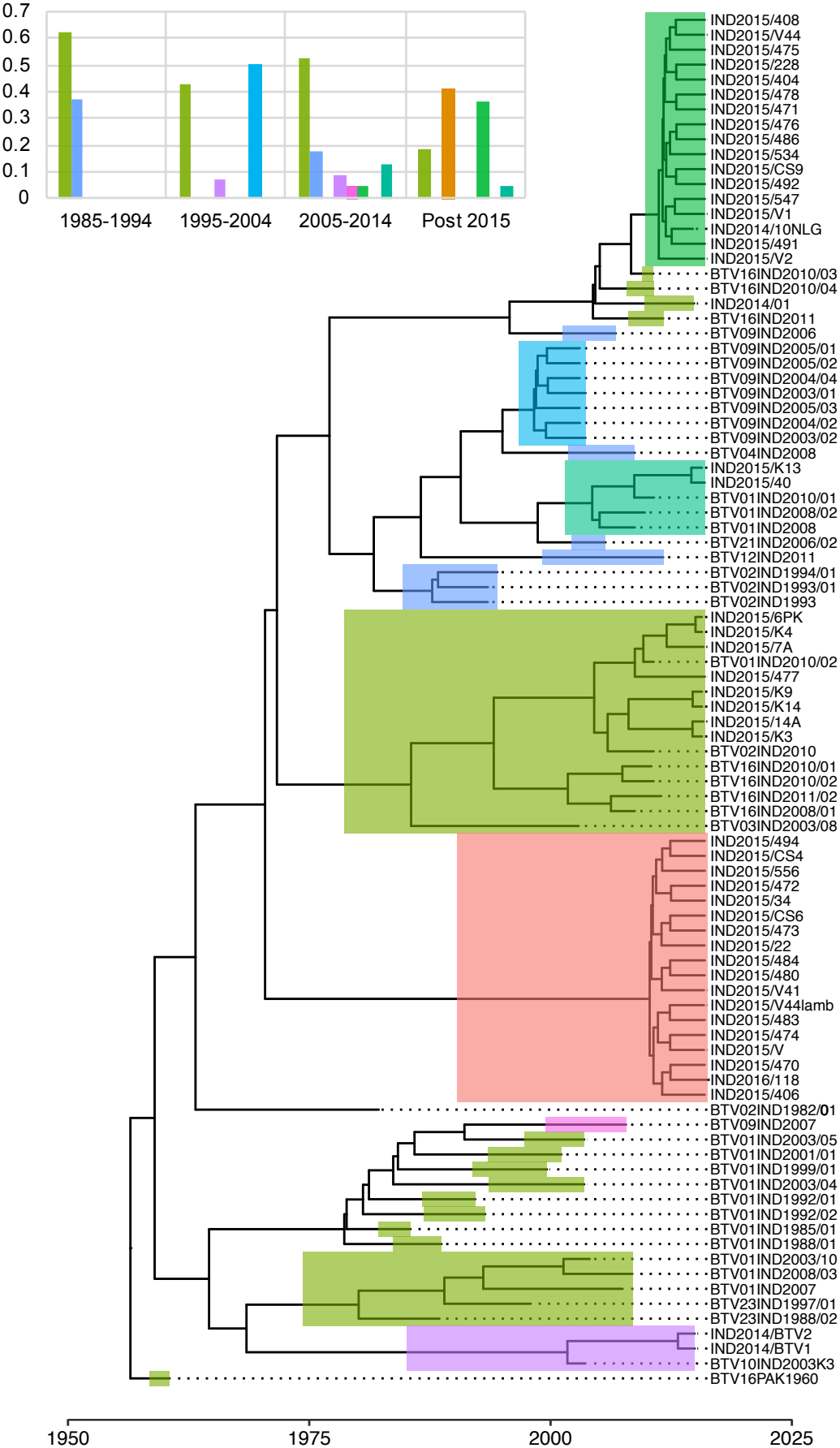

Supplement: vez027_Supplementary_Data [file vez027_supplementary_data.zip › FigureS5_Seg3_sub1.pdf]

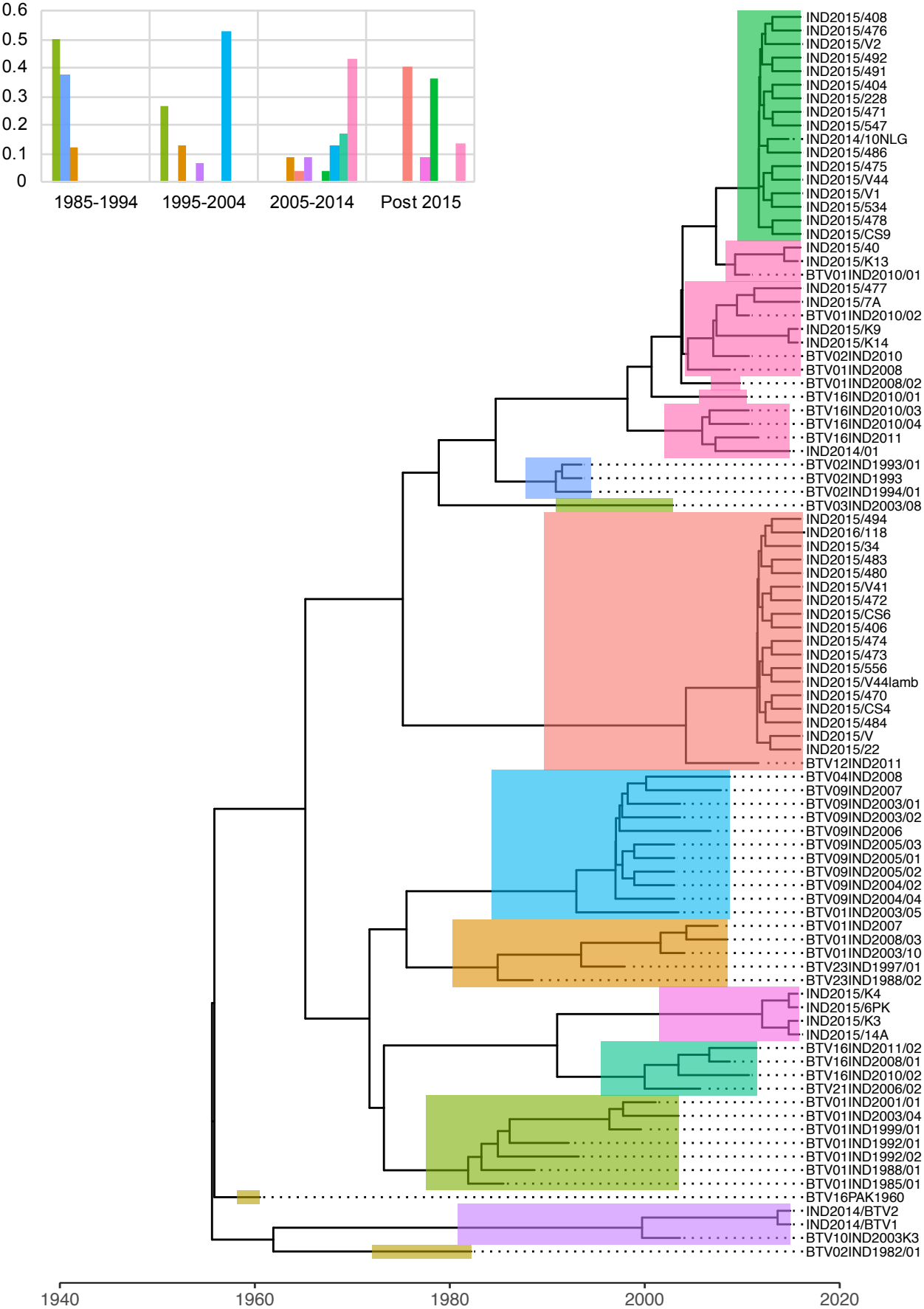

Supplement: vez027_Supplementary_Data [file vez027_supplementary_data.zip › FigureS6_Seg4_sub1.pdf]
